# Effects of Polygenic Risk Scores and Physical Activity on Blood Pressure Trajectories in Childhood, Adolescence, and Young Adulthood

**DOI:** 10.1161/CIRCGEN.125.004307

**Published:** 2026-09-24

**Authors:** Tian Xie, Shili Luo, Chris H.L. Thio, Marloes Cardol, Eva Corpeleijn, Gerton Lunter, Harold Snieder, Lifelines Cohort Study, International Consortium of Blood Pressure, Evangelos Evangelou

**Affiliations:** 1 Department of Epidemiology, University of Groningen, University Medical Center Groningen, the Netherlands (T.X., S.L., C.H.L.T., M.C., E.C., G.L., H.S.).; 2 Guangzhou National Laboratory, Guangzhou International Bio Island, China (T.X.).

**Keywords:** blood pressure, exercise, gene-environment interaction, longitudinal studies, genetic risk score

## Abstract

**BACKGROUND:**

Understanding the effects of genetics and lifestyle habits such as physical activity on blood pressure (BP) trajectories at an early age helps to improve understanding of the pathogenesis of hypertension over the life course.

**METHODS:**

BP was assessed twice (at baseline and follow-up) in prepuberty (n=1063; age 5–6 years; median follow-up, 4.72 years), puberty (n=2494; age 8–14 years; median follow-up, 3.00 years), and postpuberty (n=2549; age 15–20 years; median follow-up, 3.00 years). We calculated polygenic risk scores (PRSs) for BP based on the largest BP genome-wide association study and examined the associations of PRSs with BP trajectories in the 3 age epochs, using linear mixed models.

**RESULTS:**

One SD of PRSs was positively associated with 1.70, 1.65, and 2.92 mm Hg systolic blood pressure (SBP) level and 1.68, 0.85, and 1.18 mm Hg diastolic blood pressure (DBP) level at ages 5, 8, and 15 years, respectively. There was evidence of associations between PRSs and rates of change in SBP during puberty and DBP during postpuberty. During puberty, individuals in the highest quartile of SBP-PRS showed a 0.32 (95% CI, 0.02–0.63) mm Hg greater increase in SBP per year compared with those in the lowest quartile, while in postpuberty, those in the highest quartile of the DBP-PRS exhibited a 0.24 (95% CI, 0.02–0.45) mm Hg/y greater increase in DBP. Children who were physically active had, on average, 1.90 (95% CI, −3.77 to −0.03) mm Hg lower initial SBP than inactive children in puberty. No evidence was found for interactions of physical activity with PRS on BP levels or changes.

**CONCLUSIONS:**

Our study suggests that genetic influences on BP are evident as early as age 5 and extend to dynamic changes in SBP during puberty and DBP during postpuberty. Higher physical activity may reduce SBP levels in puberty independent of genetic susceptibility.

Elevated blood pressure (BP), with its worldwide increasing prevalence in both adults and children, is the leading contributor to global disease burden.^1,2^ A recent Mendelian randomization study in the UK Biobank showed that elevated BP is causally associated with an increased risk of cardiovascular diseases and related outcomes.^3^ Also, a large-scale analysis of randomized trials demonstrated that a 5 mm Hg reduction of systolic BP (SBP) reduced the risk of major cardiovascular events by about 10%.^4^ These studies confirm the importance of hypertension management in cardiovascular disease risk reduction.

Genetic and lifestyle factors and their interactions play a key role in BP development.^5^ The largest genome-wide association study (GWAS) of BP traits to date, in >1 million adults, has identified over 2000 independent BP signals from known loci and novel loci, in total explaining ≈7% of the variance of systolic and diastolic BP.^6^ Polygenic risk scores (PRSs) combining genetic information of BP variants have been shown to be associated with BP and hypertension in adults^6,7^ and children.^8^ These scores provide an opportunity to investigate how genetic susceptibility contributes to BP regulation across the life course.

In addition, BP tends to increase with age during childhood and adolescence.^9^ Understanding whether BP-PRSs also influence these age-related changes in BP may therefore provide insight into when genetic susceptibility to elevated BP begins to manifest. However, most previous longitudinal studies were performed in adults,^10–12^ and effects of genetic factors on longitudinal BP changes in children and adolescents remain much less clear.^13,14^

A previous prospective study showed an association of a PRS with SBP at 6 years but not with SBP changes through age 17 years, using 29 BP-associated single nucleotide polymorphisms (SNPs) to calculate the PRS.^14^ However, this approach might have been underpowered to find such effects. Recently, many more genetic markers have been identified in large-scale GWAS meta-analyses.^6,7^ In addition, methods for constructing PRSs have been developed to incorporate more information, including non-genome-wide significant SNPs and functional annotations, thereby substantially enhancing their predictive power.^6^ PRSs based on the largest GWAS to date and more advanced methods are expected to provide greater statistical power to detect genetic effects.

Physical activity (PA) is regarded as an important lifestyle factor in the prevention and treatment of hypertension in both adults and children.^15,16^ Earlier evidence from cross-sectional studies showed that interactions between PRSs and PA may play a role in the regulation of BP,^17,18^ but whether PA interacts with genetic factors for BP changes over time requires further investigation.

Moreover, genetic and PA effects on BP changes might vary during different developmental periods (eg, prepuberty, puberty, and postpuberty), but there is a lack of studies exploring such different time windows in the same study. Given that BP levels in children track into adulthood,^19,20^ understanding genetic and PA effects and their interaction on BP trajectories at an early age may help to improve our understanding of the pathogenesis of hypertension over the life course and inform preventive strategies.

Therefore, the aim of our study was to explore the effects of BP-PRSs and PA on BP trajectories in 3 age epochs: childhood, adolescence, and young adulthood. Furthermore, we investigated whether PA interacted with PRSs on BP trajectories. To this end, we calculated PRSs of BP using the largest GWAS of BP to date and assessed the associations of PRSs and PA with BP trajectories in 3 age epochs: prepuberty (5–6 years at baseline), puberty (8–14 years at baseline), and postpuberty (15–20 years at baseline), in participants of 2 prospective population-based cohorts. We focused on SBP and diastolic BP (DBP) as these traits represent the fundamental components of BP and are the primary phenotypes used in clinical risk stratification and hypertension definition.

## Methods

### Data Sharing

This study used data from 2 cohorts: the GECKO Drenthe study (Groningen Expert Center for Kids With Obesity) and the Lifelines Cohort Study and Biobank. Because of the sensitive nature of the data used in this study, requests to access the data set from qualified researchers trained in human subject confidentiality protocols may be sent to the GECKO Drenthe cohort and Lifelines cohort. Researchers who are interested in using data for research purposes can find more information about the GECKO Drenthe cohort on www.birthcohorts.net. Lifelines is a facility that is open to all researchers; information on application and data access procedures is available on https://www.lifelines.nl/researcher/how-to-apply.

### Study Population

The GECKO Drenthe study is a Dutch population-based birth cohort that studies risk factors associated with the development of overweight and obesity from birth to adulthood.^21^ The GECKO Drenthe cohort has been described in detail elsewhere.^21^ In brief, the cohort includes up to 2842 actively participating children born between April 2006 and April 2007. Data collection started from the last trimester of pregnancy, cord blood samples were collected, and monitoring of children is still ongoing. In the current study, baseline was defined as the first BP measurement (age 5–6 years), rather than the time of recruitment, and follow-up was defined as the second measurement (age 10–11 years). The current study included 1063 European children from GECKO Drenthe who have genetic data and BP measurements at baseline (5–6 years) or follow-up (10–11 years; Figure 1 shows the flowchart of study inclusions in GECKO Drenthe). Written informed consent was obtained from parents, and this study was approved by the Medical Ethics Committee of the University Medical Center Groningen in accordance with the 1975 Declaration of Helsinki, as amended in 1983.

**Figure 1.**
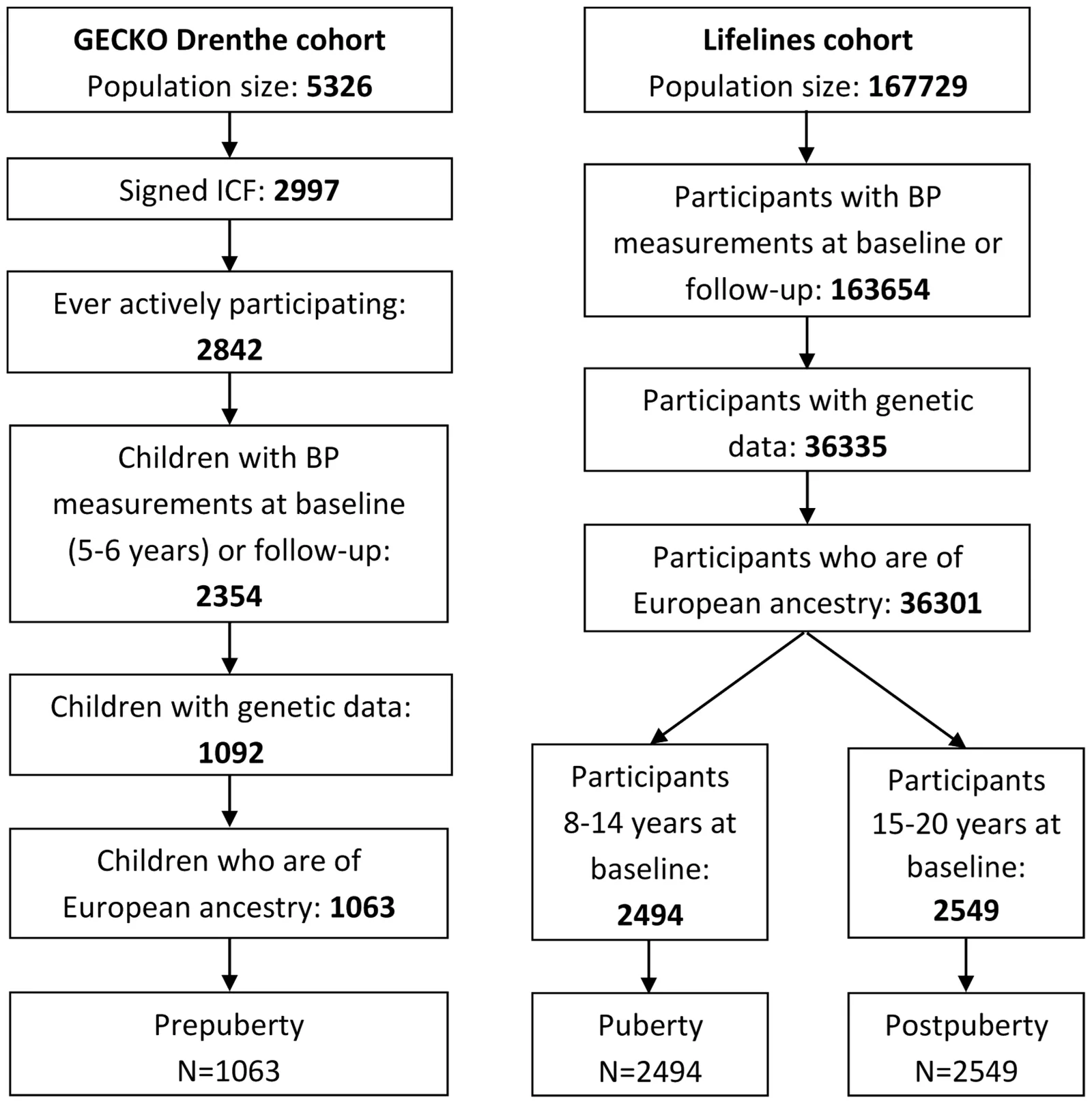
**Flowchart of the inclusion of study participants in GECKO Drenthe (Groningen Expert Center for Kids With Obesity; left) and Lifelines (right).** BP indicates blood pressure; and ICF, informed consent form.

The Lifelines Cohort Study is a large prospective population-based study performed in the northern Netherlands with a 3-generation design, aimed at investigating risk factors for multifactorial diseases.^22,23^ Details of Lifelines have been published elsewhere.^22,23^ Briefly, between 2006 and 2013, baseline data were collected for 167 729 participants, and follow-up assessments have been conducted after 3 to 4 years. We used genetic data from the first release of the University Medical Center of Groningen Genetics Lifelines Initiative project comprising genotypes of both adults and children in Lifelines. Among 36 301 European participants who have genetic data and BP data at baseline or follow-up, we selected 2 populations in 2 age epochs: 2494 children who were 8 to 14 years at baseline and 2549 individuals who were 15 to 20 years at baseline (Figure 1 shows the flowchart of study inclusions in Lifelines). These age epochs were chosen a priori and were partly based on available data in Lifelines, roughly representing puberty and postpuberty. For participants aged 8 to 11 years, written informed consent was provided by their parents or legal guardians. For participants aged 12 to 17 years, consent was obtained from both the child and at least 1 parent or legal guardian. Participants aged >18 years provided written informed consent themselves. The study was approved by the medical ethical committee of the University Medical Center Groningen, the Netherlands.

### BP Measurements

Repeated BP measurements were available for the prepuberty group from the GECKO Drenthe Cohort and for the puberty and postpuberty groups from the Lifelines Cohort. In GECKO Drenthe, BP at the age of 5 to 6 and 10 to 11 years was measured by health care professionals using a digital automatic BP monitor (iOmron M3) and an appropriate cuff size. The child was sitting for 5 minutes, then the measurements were repeated up to 3× with 1-minute intervals, and the mean SBP and DBP were calculated. Details of BP measurements in GECKO Drenthe have been described elsewhere.^24^ In Lifelines, BP was measured in all participants aged 8 years and older in a seated position during baseline and follow-up using an automated DINAMAP Monitor (PRO100 or PRO100V2) and an appropriate cuff size. Participants were requested to sit still, remain quiet, and not move their arms. BP was measured 10× at baseline and 3 to 6× during follow-up with 1-minute intervals, and then the average of the final 3 readings was recorded for SBP and DBP. In both GECKO Drenthe and Lifelines, children (<18 years) were categorized as having hypertension if their SBP or DBP was ≥95th percentile for sex, age, and height, based on the American Academy of Pediatrics 2017 guideline.^16^

### Genotyping and Imputation

In GECKO Drenthe, DNA samples were genotyped using the Illumina Global Screening Array beadchip. Samples with a call rate <95%, outliers for heterozygosity, sex mismatches, or duplicated samples were excluded. SNPs with a call rate <95%, Hardy-Weinberg equilibrium *P*<10^−^6, minor allele frequency <1%, allele frequency discordant with the 1000G reference, or duplicated SNPs were removed. Next, genetic imputation was performed through the Michigan imputation service using the Haplotype Reference Consortium panel, v1.1.^25^

In Lifelines, genome-wide genotyping was also performed using the Illumina Global Screening Array. Samples with a genotyping call rate <99%, outliers for heterozygosity and sex mismatches were excluded, as well as samples that did not show consistent information between reported familial information and observed identity-by-descent sharing with family members, and between genotypes available from this and previous studies. Variants with a genotyping call rate <99%, Hardy-Weinberg equilibrium *P* value <10^−^6 or an excess of Mendelian errors in families (>1% of the parent-offspring pairs) were removed. The genotyping data set was then imputed at the Sanger imputation server using the Haplotype Reference Consortium panel, v1.1.^25^

### PRS Calculation

Our PRS derivation was based on the most recent meta-GWAS of BP conducted in over 1 million individuals of European descent, which has identified 2103 independent BP signals (*P*<5×10^−^8) from known loci and novel loci.^6^ Importantly, PRS construction was not restricted to these significant loci. Instead, we applied the SBayesRC^26^ approach to the full set of GWAS summary statistics of SBP and DBP. SBayesRC is a Bayesian method for polygenic prediction that incorporates functional genomic annotations with high-density SNPs (>7 million variants). Specifically, it integrates 96 functional annotations from BaselineLD (v.2.2)^27^ including coding status, regulatory chromatin states, transcription factor binding sites, and other functional genomic features. By leveraging linkage disequilibrium structure and annotation-informed priors, SBayesRC improves posterior SNP effect estimation and has demonstrated better prediction accuracy in both European ancestry and trans-ancestry prediction than other PRS methods.^6,26^

For each BP trait (SBP and DBP), the SBayesRC algorithm^26^ was applied to GWAS summary statistics for each BP trait to obtain posterior SNP effect estimates for over 7 million SNPs. PRSs were then calculated in GECKO Drenthe, Lifelines puberty and postpuberty samples by multiplying posterior SNP effect sizes by the corresponding risk allele dosages and summing across all SNPs using PLINK, version 1.90. After quality control and SNP matching between posterior SNP effect sizes and target genotype data, 7 356 519 SNPs were included in the PRS calculation in Lifelines and 7 356 518 SNPs in GECKO Drenthe. The resulting PRSs were then standardized to Z scores (mean = 0, SD = 1) for analysis.

We then calculated variance explained at baseline by the PRSs in GECKO Drenthe, Lifelines puberty, and postpuberty samples, respectively. First, we generated the residuals from a regression of each BP trait against sex, age, age square, height, body mass index (BMI), and the first 10 genetic principal components; then, we fitted a second linear model for the trait residuals with each PRS. The adjusted *R*^2^ of the second model was the estimation of BP trait variance explained by each PRS.

### Physical Activity

In the GECKO Drenthe cohort, PA was measured using ActiGraph GT3X accelerometers (ActiGraph, Pensacola, FL) when children were 5 to 6 years of age. Details of PA measurements in the cohort have been described elsewhere.^28^ We defined a valid measurement as having at least 3 days with a wear time of >600 min/d. The following cutoff points were used to assess different PA intensities: sedentary time (<819 counts per minute), light PA (820–3907 counts per minute), and moderate-to-vigorous PA (>3908 counts per minute).^29^ Averages of minutes per day spent in moderate-to-vigorous PA were calculated. Furthermore, according to the World Health Organization guidelines’ recommendation of a minimum of 60 minutes of moderate-to-vigorous PA a day for children,^30^ children were classified as being active if they reached a minimum of 60 minutes of moderate-to-vigorous PA a day, and inactive otherwise. In total, 488 children with PA measurement in GECKO Drenthe were included in the analyses of PA.

In Lifelines, PA was measured through questionnaires for children who were 12 to 17 years at baseline. Children were asked, On how many days in the past week did you exercise for at least 60 minutes? Children were classified as being active if their answers were at least 5 days, and inactive otherwise. Children who were younger than 12 years or older than 17 years were not asked this question. Among 1069 eligible children in the Lifelines puberty sample, 696 answered this question and were included in the analyses of PA. In the postpuberty sample, 874 with PA data were included from a total of 898 eligible children.

### Statistical Analyses

In the 3 age epochs, linear mixed models (LMMs) were used to test associations between PRSs and the corresponding BP trait trajectories. Taking SBP for example, LMM were performed to test if the SBP-PRS was associated with increased initial SBP levels (ie, main effect of the PRS on the model’s intercept), and if it was associated with greater rates of SBP change (ie, coefficient of PRS × age term in the model) with adjustment for sex, both height and BMI residuals regressed from age, and the first 10 genetic principal components accounting for population stratification. As height and BMI were highly correlated with age, age was regressed out of height and BMI, separately, before performing LMM. We also conducted an additional analysis using a fully adjusted model in which height and BMI were included instead of their age-adjusted residuals. Age was centered around the youngest age at baseline (age minus 5 in GECKO Drenthe, age minus 8 in Lifelines puberty, and age minus 15 in Lifelines postpuberty). Then linear growth in SBP levels over time as a function of age (years) was explored, and SBP-PRS was introduced as a subject-level predictor of the variability in intercept and slope. Final models adopted random intercepts and slopes in the 3 age groups (prepuberty, puberty, postpuberty). To formally test whether associations differ across epochs, we pooled data from GECKO and Lifelines. We first added a 2-way interaction term (PRS × epoch) to examine whether the effect of PRS on initial BP differs by epoch. Next, we included a 3-way interaction term (PRS × age × epoch) to assess whether the effect of PRS on BP slope differs by epoch. To consider whether PRS effects on initial BP and BP slope differ by sex, we first included a 2-way interaction term (PRS × sex) and subsequently a 3-way interaction term (PRS × age × sex) to the original model. In addition, we performed analysis by including a 2-way interaction term (age × sex) in the original model to assess whether sex-specific age effects would change previous results. Two types of models were built: the first included PRS as a continuous variable (standardized PRS), the second used PRS as a categorical variable (quartiles). The model assumptions were assessed and are presented in Figures S1 through S12.

In the subsets of individuals who had PA data (N=488 in prepuberty, 696 in puberty, and 874 in postpuberty), we explored associations between PA and SBP and DBP trajectories using LMM. PA was included in the models as a binary variable (active versus inactive). We additionally conducted analyses using the original continuous (GECKO) and ordinal (Lifelines) PA measures within each cohort. In the first step (model 1), PA was introduced as a subject-level predictor of the variability in intercept and slope of each BP trait with adjustment for the PRS, sex, height residual regressed from age, and the first 10 genetic principal components (season of PA measurement and mean wear time were also adjusted in GECKO Drenthe). In the second step (model 2), BMI residual regressed from age was further adjusted. In the third step (model 3), the interaction of PA and the PRS was added in the model as subject-level predictors of the variability in intercept and slope of each BP trait. *P*<0.05 was considered statistically significant.

Finally, we performed additional analyses using generalized estimating equations,^31^ with clustering at the individual level, to check the robustness of the results. All statistical analyses were performed in R, version 4.4.0^32^ with package nlme^33^ for mixed models and geepack^34^ for generalized estimating equation.

## Results

### Basic Characteristics

Our study included 1063 participants from GECKO Drenthe in the prepuberty sample, as well as 2494 and 2549 participants from Lifelines in the puberty, and postpuberty samples, respectively, who had at least 1 BP measurement. There were 936, 1943, and 1488 participants in the prepuberty, puberty, and postpuberty groups at follow-up, respectively. Table 1 shows baseline and follow-up characteristics for these 3 samples. The mean age at baseline was 5.85, 10.97, and 17.81 years, with median follow-up duration of 4.72, 3.00, and 3.00 years for prepuberty, puberty, and postpuberty epochs, respectively. Variance explained by the PRSs ranged from 4.52% to 7.33% for SBP and from 3.30% to 4.91% for DBP in the 3 age epochs, with the largest variance explained in postpuberty. A subset of 488, 696, and 874 individuals who had PA data were used in the analyses of PA effects on BP for each period, respectively, and ≈50% of them were active.

**Table 1.**
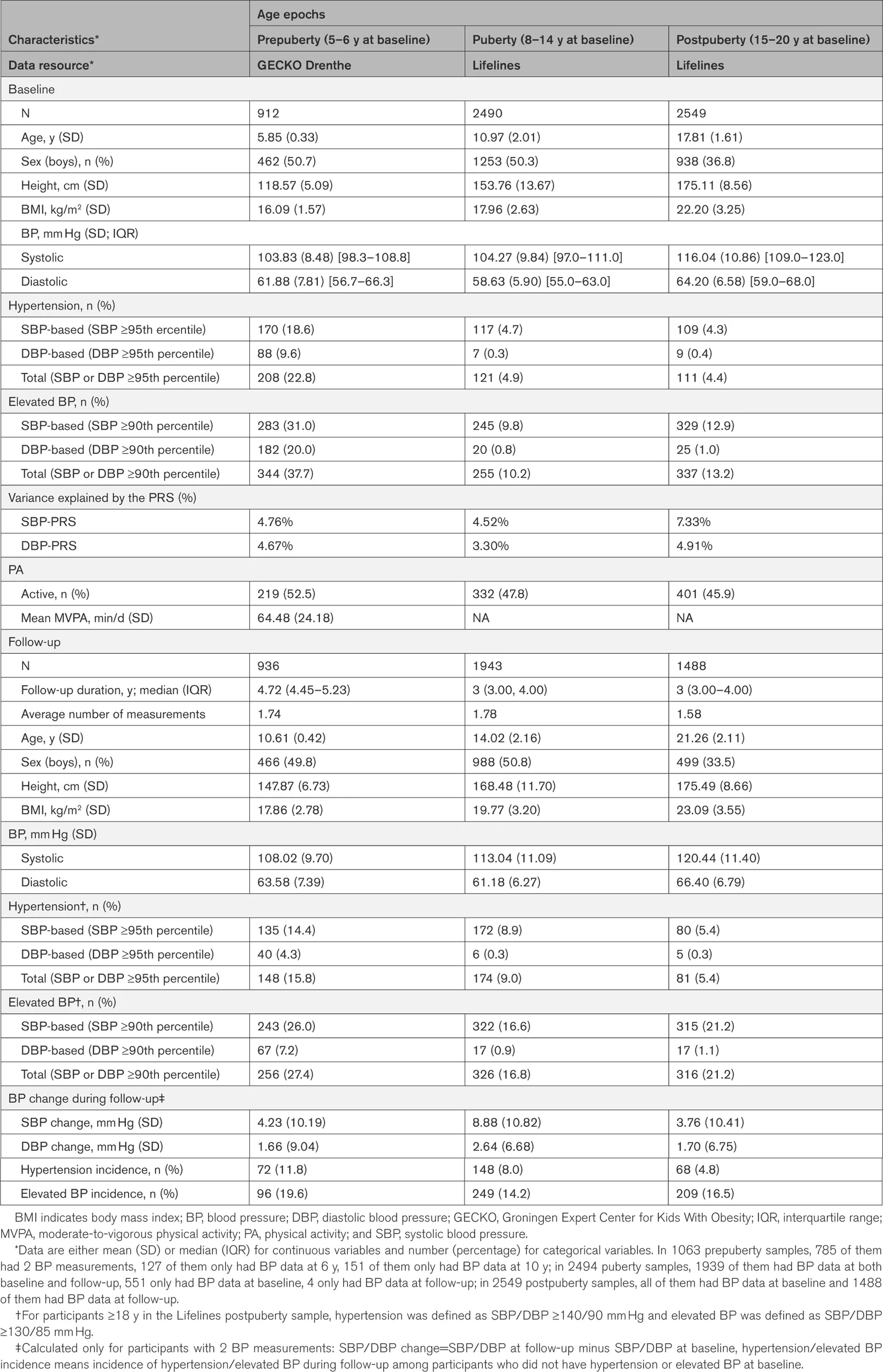
Characteristics of the Samples of the 3 Different Age Epochs

### PRSs Effects on BP Levels and Changes in 3 Age Epochs

Table 2 presents the fixed effect estimates for the PRS main effect and its interaction with age on BP trajectories in the 3 age epochs. Figure 2 illustrates the changes in mean predicted values of BP with age in every PRS quartile.

**Table 2.**
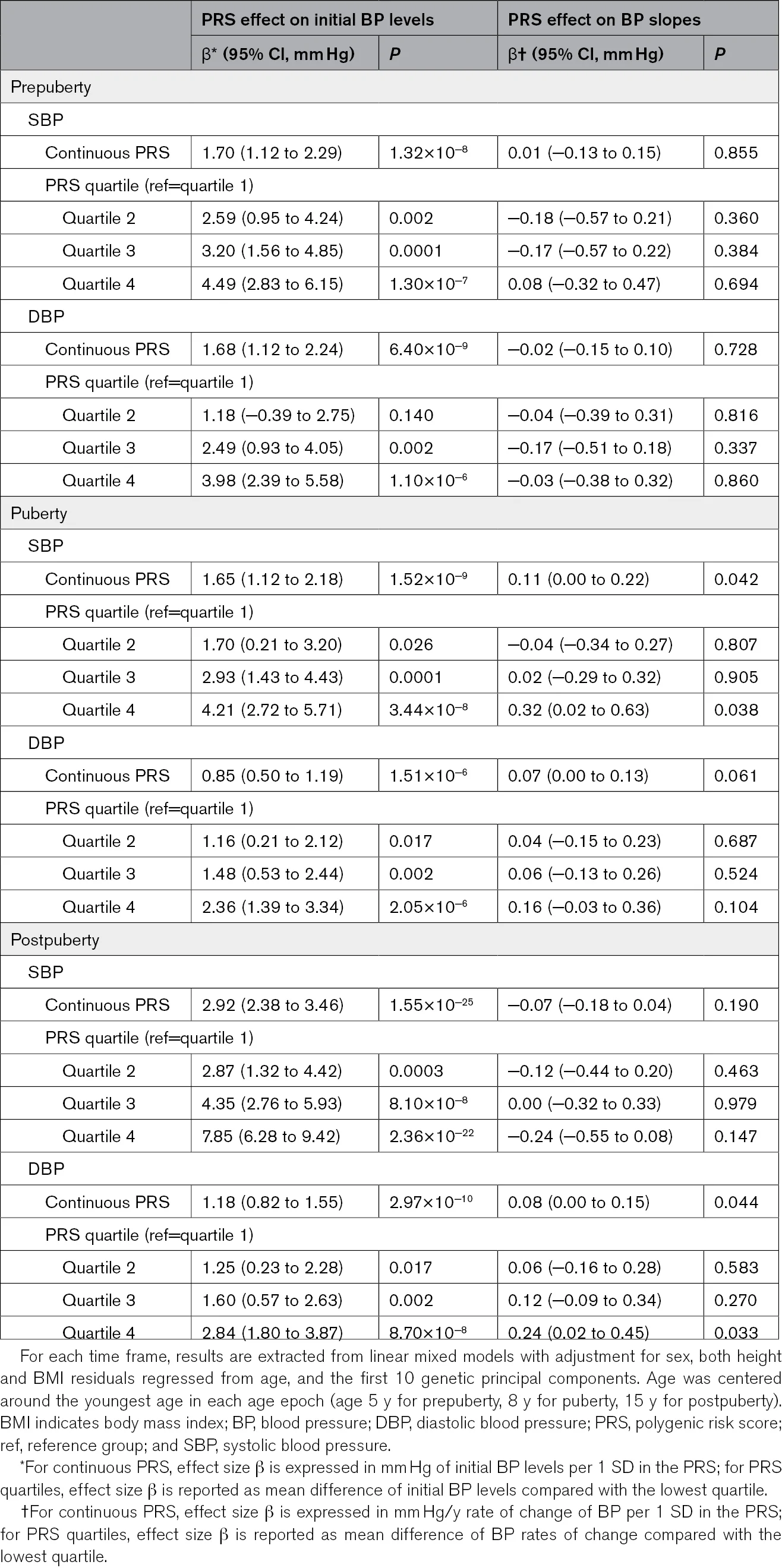
PRS Effects on BP Trajectories in the 3 Age Epochs

**Figure 2.**
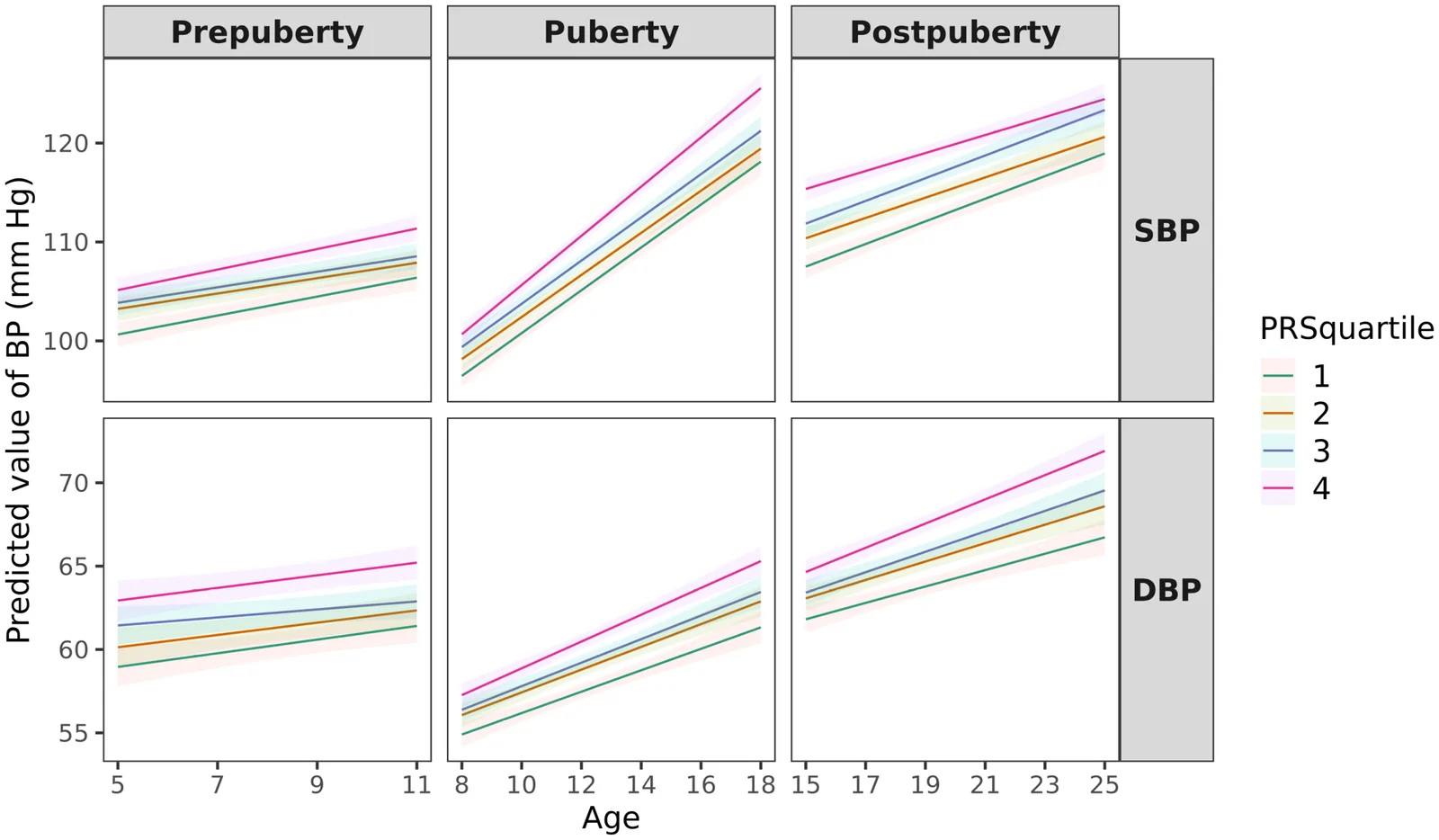
**Mean predicted value of blood pressure (BP; mm Hg) with age for individuals at each quartile of polygenic risk score (PRS) of the corresponding BP trait.** PRS quartile 1 represents the lowest 25% of the PRS, and quartile 4 represents the highest 25%. Error bars represent 95% CI. DBP indicates diastolic blood pressure; and SBP, systolic blood pressure.

In each age epoch, BP-PRSs had significant effects on initial BP levels (ie, average BP at the youngest age in each age epoch: 5 years in prepuberty, 8 years in puberty, and 15 years in postpuberty). For SBP, each SD increase in SBP-PRS was associated with 1.70, 1.65, and 2.92 mm Hg initial SBP (ie, at 5, 8, and 15 years) in prepuberty, puberty, and postpuberty, respectively. The association was stronger in postpuberty than in prepuberty (β, 0.97 [95% CI, 0.40–1.54]; *P*=0.001; Table S1). Compared with those in the lowest quartile of SBP-PRS, individuals in the highest quartile had, on average, 4.49, 4.21, and 7.85 mm Hg higher SBP at ages 5, 8, and 15 years, respectively. For DBP, each SD increase in DBP-PRS was associated with 1.68, 0.85, and 1.18 mm Hg DBP at 5, 8, and 15 years old, respectively. The association was weaker in puberty compared with prepuberty (β, −0.39 [95% CI, −0.78 to −0.01]; *P*=0.047; Table S1). Children in the highest DBP-PRS quartile had, on average, 3.98, 2.36, and 2.84 mm Hg higher initial DBP compared with those in the lowest quartile in prepuberty, puberty, and postpuberty, respectively.

For associations between PRSs and rates of change in BP, PRSs were found to be associated with faster BP growth rates in puberty for SBP and in postpuberty for DBP. Specifically, an SD increase in the continuous SBP-PRS showed a faster SBP growth rate (0.11 mm Hg/y; *P*=0.04). Individuals in the highest quartile had a 0.32 mm Hg/y faster rate of SBP increase (*P*=0.04) compared with those in the lowest SBP-PRS quartile. As children in the highest SBP-PRS quartile already had higher SBP at age 8 years and their SBP progressed faster, the gap between those in the highest and lowest SBP-PRS quartiles became wider during puberty, but this trend did not continue into postpuberty (Figure 2). For DBP, an SD increase in PRS was associated with a 0.08 mm Hg/y faster DBP growth rate in postpuberty (*P*=0.04). Compared with those in the lowest DBP-PRS quartile, individuals in the highest quartile already showed signs (although nonsignificant) of a faster DBP rate of increase in puberty, and this became more evident in postpuberty (β, 0.24 mm Hg/y; *P*=0.03; Figure 2). However, there was no strong statistical evidence that the PRS effect on BP slope differed between epochs (Table S2). For sex-specific effects, we found no evidence that the association between PRS and BP slope differed by sex (Table S3). In addition, we found that BP slopes differed between boys and girls in the 3 age epochs (except for SBP slope in prepuberty; Table S4). However, including the age × sex interaction term yielded nearly identical estimates for PRS and the PRS × age interaction compared with the model that did not include it (Table S5). Using generalized estimating equations with clustering at the individual level yielded results highly consistent with those from the LMM. The point estimates were nearly identical, and the corresponding CIs were similar (Tables S6 and S7). In additional analysis that used height and BMI instead of their age-adjusted residuals, the coefficients, standard errors, and *P* values for the terms of interest (PRS and PRS × age) remained unchanged compared with those from the residual-based model (Table S8).

### PA Effects

Table 3 shows results of PA effects and interactions of PA and PRSs on SBP trajectories in 3 age epochs, and Table S9 shows results for DBP. For SBP, PA was only found to be associated with initial SBP level in puberty. Children who were active had, on average, 1.90 mm Hg lower SBP at age 8 than inactive children (*P*=0.046). After additional adjustment for BMI, the effect was slightly attenuated (β, −1.78 mm Hg; *P*=0.060) and became borderline significant. There was little evidence that PA was associated with initial DBP levels in any age epoch (prepuberty: β, 0.22 [95% CI, −1.52 to 1.96]; puberty: β, −0.82 [95% CI, −1.96 to 0.33]; postpuberty: β, −0.75 [95% CI, −1.75 to 0.26]; Table S9). We did not find evidence that PA was associated with BP slope in any epoch (SBP: prepuberty, β, 0.04 [95% CI, −0.38 to 0.45]; puberty, β, 0.10 [95% CI, −0.48 to 0.68]; postpuberty, β, 0.33 [95% CI, −0.18 to 0.85]; DBP: prepuberty, β, 0.07 [95% CI, −0.30 to 0.44]; puberty, β, 0.18 [95% CI, −0.18 to 0.53]; postpuberty, β, 0.18 [95% CI, −0.17 to 0.54]; Table 3; Table S9). In addition, there was little evidence of interactions between PA and BP-PRSs on initial BP levels or BP slopes in any age epoch (Table 3; Table S9). The results from generalized estimating equations were highly consistent with those obtained from LMM analysis (Tables S10 and S11). In additional analysis that included height and BMI instead of their age-adjusted residuals, the results remained identical compared with those from the residual-based model (Tables S12 and S13). The results from analysis using the original continuous or ordinal PA measures are shown in Tables S14 and S15.

**Table 3.**
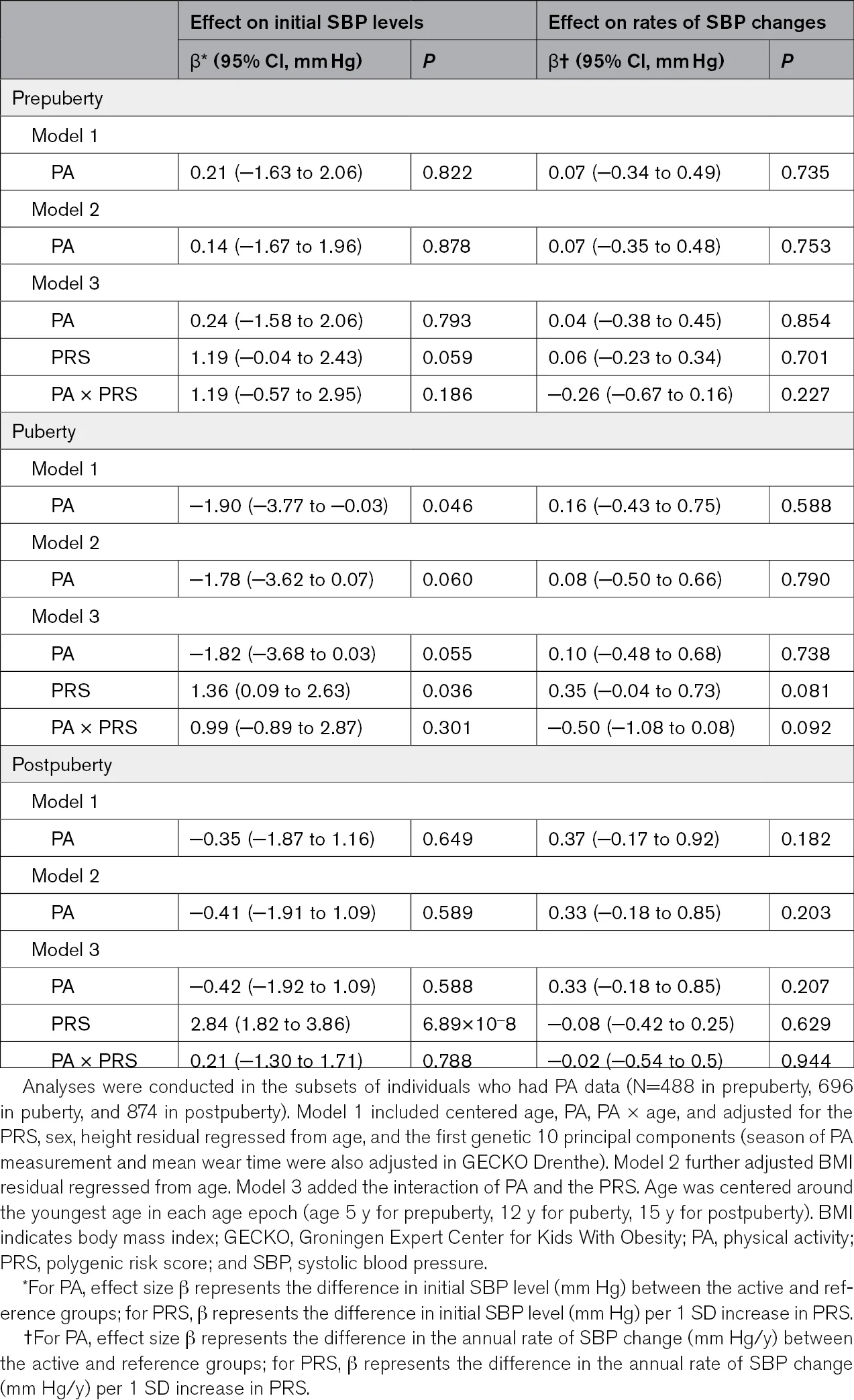
PA Effects on SBP Trajectories in the 3 Age Epochs

## Discussion

In this study, we explored the effects of BP-PRSs, PA, and their interactions on BP trajectories in 3 age epochs covering childhood, adolescence, and early adulthood. Our results showed that BP-PRSs had significant effects on initial levels of corresponding BP traits in each age epoch. PRSs were significantly associated with faster BP growth rates in puberty for SBP and in postpuberty for DBP. PA was only found to be associated with initial SBP level in puberty.

Consistent with previous literature,^8,13^ our findings demonstrated that higher PRSs were associated with elevated BP levels starting from as young as 5 years and that the effects continued into adolescence and early adulthood. We found each SD increase in SBP-PRS was associated with an average increase of ≈1 to 3 mm Hg in SBP across the 3 age epochs. Compared with individuals in the lowest SBP-PRS quartile, those in the highest quartile had ≈4 to 8 mm Hg higher SBP levels across epochs. Although these effect sizes may appear modest, individuals with higher genetic risk may reach clinically relevant BP thresholds earlier than those with lower genetic risk,^35^ potentially leading to greater cumulative exposure to elevated BP (eg, mm Hg × years). Increased cumulative BP exposure has been associated with a higher risk of cardiovascular and cerebrovascular outcomes.^36^ At the population level, even small shifts in BP distribution can have substantial public health implications. For example, a 2 mm Hg lower usual SBP is associated with ≈10% lower stroke mortality and about 7% lower mortality from ischemic heart disease or other vascular causes in middle age.^37^ Although PRSs in our study were generated based on a meta-GWAS of BP in adults, these adult-based PRSs had detectable effects on BP already in young children. Therefore, adult-based PRSs for BP may help to identify individuals predisposed to higher BP at an early age. However, the variance explained by PRSs in the 3 age epochs (4.52%–7.33% for SBP and 3.30%–4.91% for DBP) was smaller than the 11% to 12% of variance explained in adults.^6^ This may be due to age-dependent genetic effects: different genes may influence the phenotypes at different ages, or the magnitude of effects of the same genes may change over time.^38,39^

BP tends to increase with age in childhood and adolescence; possible reasons may include natural progression of aging, increase in height (ie, growth), adiposity increases, lifestyle changes, or other unexplored mechanisms.^9^ In this study, we explored if genetic factors also contribute to the rate of change in BP in childhood and adolescence. We found that PRSs are associated with faster BP growth, but only in a specific time window (ie, puberty for SBP and postpuberty for DBP); however, there was no strong statistical evidence that these associations differed across age epochs. The associations were independent of growth and adiposity increases because height and BMI were adjusted in the models. Several previous studies observed associations between BP-PRSs and BP changes in middle-aged populations.^10–12^ For instance, Fava et al^10^ showed that an increase of 1 SD of the PRS including 29 BP-related SNPs was associated with an increase of 0.033 mm Hg/y for SBP and 0.023 mm Hg/y for DBP in middle-aged Swedes. But this association was not previously detected in children or adolescents. Howe et al reported little to weak evidence of an association between a 29-SNP allelic score and SBP change across childhood and adolescence (age 6–11 y: β, 0.002, SE, 0.009; *P*=0.862; 11–15 y: β, 0.023, SE, 0.012; *P*=0.062; 15–17 y: β, −0.010, SE, 0.020; *P*=0.619).^14^ One probable reason is that they used a PRS of only 29 SNPs, which explained a mere 0.2% of variance in SBP at 17 years of age. As sample sizes of BP GWASs have recently expanded dramatically, leading to an increasing number of identified SNPs and more accurate effect sizes, our PRSs using the largest GWAS findings could explain at least 4.52% of SBP variance. Therefore, we had much stronger scores and were able to observe effects of PRSs on BP slopes. Findings of Howe et al showed similar patterns to our data, although their results were not significant. They found weak evidence of an association between the allelic score and SBP change between 11 and 15 years of age, but not between 6 and 11 years of age and between 15 and 17 years of age.^14^ Thus, they also showed the allelic score may be related to SBP change in a specific time window, and this time window largely overlaps with ours.

The observed associations between PRSs and BP changes, which appeared evident during puberty for SBP and postpuberty for DBP, may reflect developmental differences in SBP and DBP trajectories at early ages. Labarthe et al found that SBP and DBP were distinct in patterns of change with age: SBP rises through childhood to a peak at around age 15/16 years of age and then remains stable, while DBP appears to increase continuously from 8 to 18 years of age.^40^ In addition, Shankar et al found that the increases in SBP (adjusted for height and BMI) were much greater during the pubertal growth period (9–14 years of age for females and 11–16 years of age for males) than either before or after, but this phenomenon was less evident for DBP.^41^ Another study using longitudinal data from UK cohorts also showed a rapid increase in SBP coinciding with peak adolescent growth. Although we did not find much evidence that the association between PRS and BP slope differed across 3 age epochs, our results demonstrate that PRSs are associated with BP changes during specific developmental periods, broadly aligning with known age-related SBP and DBP trajectories.

It is worth mentioning that the GWAS we used to calculate PRSs was cross-sectional and may not have captured all genetic variants associated with BP changes or obtained appropriate effect sizes of the associations. The only meta-GWAS for longitudinal BP traits^42^ to date was performed in 33 720 trans-ethnic individuals and identified several novel loci associated with BP changes that were not detected in cross-sectional BP GWASs. This suggests that there may be differences in genetic architecture and underlying biological mechanisms between cross-sectional BP and BP changes.^42^ Future longitudinal GWASs with larger sample sizes may identify more genetic variants that impact BP trajectories and help to improve genomic prediction of BP changes.

For PA, we observed that active PA was associated with lower initial SBP level in puberty. However, the effect size and statistical significance attenuated after adjusting for BMI, which suggests that BMI may partly mediate the relationship between PA and SBP, indicating that the protective effect of PA on BP in puberty may partly operate through its impact on body composition. A systematic review summarized that PA was associated with BP in around half of the studies in youth.^43^ The lack of consistency in previous research along with our findings may suggest PA effects on BP in specific time windows such as puberty or late adolescence.^44^

PA was not significantly associated with BP slopes in any age epoch. Across epochs, the estimated effects of PA on rates of SBP change were small, with CIs ranging from −0.50 to 0.92 mm Hg. Epidemiological evidence suggests that even modest differences (eg, 2 mm Hg) in BP are associated with meaningful differences in cardiovascular risk.^37^ These CIs encompass effect sizes of potential practical relevance, indicating that our findings reflect an absence of evidence rather than evidence of no effect. Thus, we cannot entirely exclude the possibility of reverse causality between PA and BP. However, unlike for adiposity, it is unlikely that high BP leads to lower levels of PA, whereas it is biologically plausible that PA affects BP through several potential pathways.^45^

Previous studies have suggested that the effect of PA on cardiovascular outcomes may differ according to genetic susceptibility.^46^ Individuals with higher BP-PRS are at increased risk of hypertension; however, higher levels of PA may attenuate this genetic risk.^47^ Xi also reported that genetic risk for higher BP was evident only among physically inactive children when compared with physically active children.^17^ In the present study, we found no evidence showing a significant interaction between PA and BP-PRS. Differences in the measurement and categorization of PA and genetic susceptibility (single SNP variants and 5-SNP multi-SNP PRS in Xi et al versus full genome PRS in our study), as well as differences in population characteristics and the range of PA levels, may have contributed to the inconsistent findings. Interaction effects are typically smaller in magnitude than main effects and therefore require much larger sample sizes to detect.^48^ In our study, the smaller sample sizes within the 3 age epochs with PA data (n=488, 696, and 874, respectively) may have limited our ability to detect such interactions. Furthermore, the identification of gene-environment interactions is sensitive to measurement error in environmental exposures.^49^ In this study, PA was partly assessed by questionnaires, which may not accurately capture participants’ true activity levels and could have further attenuated the observed interaction effects.

Although Moraes et al^18^ found that performing at least 60 min/d of PA can attenuate the deleterious effect of the Nitric Oxide Synthase 3 variant on SBP in European adolescents, our study did not detect any statistically significant interactions between PRSs and PA on BP changes. However, they explored each known BP-associated variant instead of an allelic score combining all known variants. It is possible that gene-PA interactions only exist in specific variants associated with BP, although the underlying biological mechanisms still need further investigation. Taken together, PA at baseline may not slow down future BP increases in youth, but higher PA may reduce SBP levels independently of genetic factors and thus put children on a lower (but not slower) SBP trajectory in puberty.

We observed a relatively high prevalence of BP ≥90th and ≥95th percentiles in GECKO, largely due to the use of the 2017 American Academy of Pediatrics guideline,^16^ which is based on normative BP references from normal-weight children and therefore applies lower percentile thresholds than earlier guidelines (eg, the 2004 Fourth Report^50^). This results in higher estimated prevalences, particularly among children with overweight or obesity. In our previously published work,^24^ we demonstrated that applying the 2004 criteria yielded substantially lower hypertension prevalence (11.4%) in GECKO. In addition, compared with some other Dutch cohorts, GECKO participants have a higher prevalence of overweight/obesity and less favorable cardiometabolic profiles, which likely contributes to higher BP distributions. These factors should be considered when interpreting absolute prevalence estimates; however, our prior work has demonstrated that associations between early life determinants and BP are generally consistent across cohorts despite differences in baseline BP levels, suggesting that such differences are unlikely to materially influence the interpretation of association results.^24^

Besides the limitations mentioned above, there are some other limitations to our study. First, we had only 2 BP measurements in GECKO Drenthe and Lifelines, so we were not able to model nonlinear changes of BP. In addition, BP was measured using different protocols across the 2 cohorts. This measurement heterogeneity may reduce the comparability of effect estimates across epochs and contribute to increased variability in slope estimates, potentially biasing estimates toward the null. Second, using cross-sectional GWAS to build PRS may underestimate genetic effects on BP slopes. PRS incorporating variants identified in longitudinal GWASs may predict BP changes better. Third, PA was assessed using different methods across cohorts, with accelerometer-based measurements in GECKO and questionnaire-based assessments in Lifelines. This methodological heterogeneity may affect the comparability of PA across cohorts. Self-reported PA is generally less accurate than objective measurements for estimating the intensity and duration of activity and is susceptible to recall and reporting bias.^51^ There were no parents or guardians present to verify the children’s self-reported PA in Lifelines. Previous evidence suggests that adolescents tend to overestimate their PA levels.^52^ Therefore, self-reported activity in Lifelines may be overestimated. Fourth, we used chronological age as a proxy for pubertal stage, as direct measures of biological maturation (eg, Tanner stages) were not available in GECKO and Lifelines. This approach can result in misclassification, as children of the same chronological age can differ in their pubertal development.^53^ Previous evidence indicates children who experience puberty earlier than their peers tend to have higher BP.^54^ Consequently, this misclassification may introduce within-group variability in BP and bias the estimated associations. Finally, we used meta-GWAS results of European ancestry and applied PRS in children/adolescents of the same ancestry. Previous studies have demonstrated that PRSs derived from European ancestry GWAS exhibit reduced predictive performance in non-European populations, likely due to differences in causal variants, linkage disequilibrium structure, allele frequencies, and effect size distributions across populations.^55^ Therefore, further studies may be needed to determine if our results can be generalized to other ancestries. Despite these limitations, our research contributes to the understanding of genetic influence on BP trajectories in a life course perspective. Longitudinal data on BP in children permitted us to explore genetic and PA effects on both BP levels and changes at an early age. In addition, combining 2 prospective population-based cohorts enabled us to assess the associations between PRSs/PA and BP trajectories in 3 age periods, providing opportunities for identifying developmental periods during which genetic influences on BP changes may be more detectable. Finally, we made use of the largest GWAS of BP and more advanced methods to calculate PRS; therefore, we had more accurate scores than previous studies.

In conclusion, the present study shows that the influence of PRSs on BP levels starts from as young as 5 years of age and extends to systolic and diastolic BP changes during puberty and postpuberty, respectively. Higher PA appears to lower BP levels independently of genetic risk. These findings provide new insights into hypertension pathogenesis and help to inform prevention strategies implemented in early life to reduce BP levels attained in adulthood.

## Article Information

### Acknowledgments

The authors are grateful to the families who took part in the Groningen Expert Center for Kids With Obesity (GECKO) Drenthe study, the midwives, gynecologists, nurses, general practitioners, and all health professionals at Preventive Child Healthcare Drenthe for their help in recruitment and measurements, and the GECKO Drenthe study team. The authors wish to acknowledge the services of the Lifelines Cohort Study, the contributing research centers delivering data to Lifelines, and all the study participants. The authors thank the International Consortium for Blood Pressure Consortium for providing summary statistics.

### Disclosures

None.

### Supplemental Material

Tables S1–S15

Figures S1–S12

Authors & Contributors from the International Consortium of Blood Pressure (ICBP)

## Supplementary Material
