# Supplementary material for: Effects of Polygenic Risk Scores and Physical Activity on Blood Pressure Trajectories in Childhood, Adolescence, and Young Adulthood
Source: Circ Genom Precis Med. 2026 Sep 24;19(5):e004307. doi: 10.1161/CIRCGEN.125.004307 (PMC13686632; doi:10.1161/CIRCGEN.125.004307)
Supplement: Supplementary file 1 [file hcg-19-e004307-s001.pdf]

SUPPLEMENTAL MATERIAL

Table S1. Differences in the association between PRS and initial BP across age epochs

	SBP		DBP	
	β (95%CI, mmHg)	*P*	β (95%CI, mmHg)	*P*
**Continuous PRS (ref=Epoch_prepuberty)**				
Epoch_puberty × PRS	0.45 (-0.12, 1.02)	0.124	-0.39 (-0.78, -0.01)	0.047
Epoch_postpuberty × PRS	0.97 (0.40, 1.54)	0.001	-0.03 (-0.41, 0.36)	0.894
**PRS quartile (ref=Epoch_prepuberty PRS_Q1)**				
Epoch_puberty × PRS_Q2	-0.32 (-1.95, 1.31)	0.698	0.39 (-0.70, 1.48)	0.485
Epoch_puberty × PRS_Q3	0.43 (-1.20, 2.06)	0.601	-0.11 (-1.20, 0.98)	0.841
Epoch_puberty × PRS_Q4	0.94 (-0.69, 2.58)	0.258	-0.60 (-1.70, 0.49)	0.282
Epoch_postpuberty × PRS_Q2	0.43 (-1.20, 2.07)	0.603	0.54 (-0.55, 1.64)	0.332
Epoch_postpuberty × PRS_Q3	1.67 (0.03, 3.31)	0.046	0.21 (-0.89, 1.31)	0.708
Epoch_postpuberty × PRS_Q4	2.37 (0.73, 4.02)	0.005	0.12 (-0.97, 1.22)	0.824

PRS, polygenic risk score; BP, blood pressure; SBP, systolic blood pressure; DBP, diastolic blood pressure; CI, confidence interval; ref, reference group. Models included PRS, Epoch, PRS × Epoch, centered age, sex, age-adjusted height residual, age-adjusted BMI residual, and genetic principal components (PC1–PC10).

Table S2. Differences in the association between PRS and BP slope across age epochs

	SBP		DBP	
	β (95%CI, mmHg)	*P*	β (95%CI, mmHg)	*P*
**Continuous PRS (ref=Epoch_prepuberty)**				
Epoch_puberty × age × PRS	0.11 (-0.07, 0.29)	0.242	0.10 (-0.03, 0.23)	0.122
Epoch_postpuberty × age × PRS	-0.08 (-0.26, 0.10)	0.366	0.11 (-0.02, 0.24)	0.099
**PRS quartile (ref=Epoch_prepuberty PRS_Q1)**				
Epoch_puberty × age × PRS_Q2	0.11 (-0.39, 0.62)	0.662	0.11 (-0.25, 0.47)	0.557
Epoch_puberty × age × PRS_Q3	0.21 (-0.29, 0.72)	0.410	0.25 (-0.12, 0.61)	0.183
Epoch_puberty × age × PRS_Q4	0.25 (-0.25, 0.76)	0.327	0.24 (-0.12, 0.60)	0.189
Epoch_postpuberty × age × PRS_Q2	0.07 (-0.45, 0.58)	0.798	0.14 (-0.23, 0.51)	0.452
Epoch_postpuberty × age × PRS_Q3	0.19 (-0.33, 0.71)	0.477	0.31 (-0.05, 0.68)	0.095
Epoch_postpuberty × age × PRS_Q4	-0.29 (-0.81, 0.23)	0.271	0.30 (-0.07, 0.67)	0.106

PRS, polygenic risk score; BP, blood pressure; SBP, systolic blood pressure; DBP, diastolic blood pressure; CI, confidence interval; ref, reference group. Models included PRS, Epoch, age × PRS, age × Epoch, PRS × Epoch, age × PRS × Epoch, centered age, sex, age-adjusted height residual, age-adjusted BMI residual, and genetic principal components (PC1–PC10).

Table S3. Sex-Specific Effects of PRS on initial BP and BP Slope in three age epochs

	Sex-Specific Effects of PRS on initial BP			Sex-Specific Effects of PRS on BP slope	
	β * (95% CI, mmHg)	*P*		β † (95% CI, mmHg)	*P*
**Prepuberty**					
SBP					
Continuous PRS	-0.16 (-1.01, 0.69)	0.713		0.03 (-0.24, 0.31)	0.810
PRS quartile (ref=boy and quartile 1)					
Quartile 2	2.22 (-0.20, 4.65)	0.073		-0.36 (-1.14, 0.42)	0.364
Quartile 3	0.23 (-2.21, 2.66)	0.854		-0.41 (-1.19, 0.37)	0.306
Quartile 4	0.56 (-1.89, 3.00)	0.654		-0.09 (-0.87, 0.70)	0.828
DBP					
Continuous PRS	-0.81 (-1.53, -0.10)	0.026		0.11 (-0.13, 0.36)	0.366
PRS quartile (ref=boy and quartile 1)					
Quartile 2	-0.08 (-2.13, 1.96)	0.937		0.23 (-0.46, 0.93)	0.512
Quartile 3	-1.17 (-3.20, 0.86)	0.258		0.53 (-0.16, 1.22)	0.131
Quartile 4	-2.86 (-4.90, -0.82)	0.006		0.28 (-0.41, 0.98)	0.427
**Puberty**					
SBP					
Continuous PRS	0.13 (-0.49, 0.75)	0.680		0.08 (-0.14, 0.30)	0.464
PRS quartile (ref=boy and quartile 1)					
Quartile 2	0.32 (-1.42, 2.05)	0.721		-0.19 (-0.80, 0.42)	0.537
Quartile 3	-0.20 (-1.94, 1.53)	0.817		0.14 (-0.47, 0.75)	0.652
Quartile 4	0.46 (-1.27, 2.19)	0.601		0.10 (-0.50, 0.71)	0.737
DBP					
Continuous PRS	0.00 (-0.38, 0.38)	0.999		0.12 (-0.02, 0.26)	0.087
PRS quartile (ref=boy and quartile 1)					
Quartile 2	0.28 (-0.80, 1.37)	0.610		-0.01 (-0.40, 0.37)	0.940
Quartile 3	-0.05 (-1.13, 1.04)	0.932		0.08 (-0.31, 0.48)	0.673
Quartile 4	0.32 (-0.76, 1.41)	0.557		0.20 (-0.19, 0.59)	0.314
**Postpuberty**					
SBP					
Continuous PRS	0.32 (-0.37, 1.02)	0.362		-0.22 (-0.45, 0.01)	0.057

PRS quartile (ref=boy and quartile 1)					
Quartile 2	1.51 (-0.53, 3.54)	0.147		-0.06 (-0.73, 0.61)	0.860
Quartile 3	1.18 (-0.85, 3.21)	0.255		-0.43 (-1.10, 0.25)	0.215
Quartile 4	1.59 (-0.46, 3.63)	0.129		-0.32 (-0.99, 0.36)	0.360
DBP					
Continuous PRS	0.11 (-0.34, 0.56)	0.640		0.00 (-0.16, 0.15)	0.951
PRS quartile (ref=boy and quartile 1)					
Quartile 2	-0.27 (-1.57, 1.04)	0.689		-0.16 (-0.61, 0.29)	0.487
Quartile 3	-0.12 (-1.43, 1.18)	0.853		-0.28 (-0.74, 0.17)	0.216
Quartile 4	0.29 (-1.02, 1.60)	0.666		0.05 (-0.40, 0.50)	0.828

CI, confidence interval; SBP, systolic blood pressure; DBP, diastolic blood pressure; PRS, polygenic risk score; ref, reference group.

* Sex-specific effects of PRS on initial BP were derived from the sex × PRS interaction term in a linear mixed model including centered age, sex, PRS, age × PRS, and adjusted for age-adjusted height residual, age-adjusted BMI residual, and the first 10 genetic principal components. Age was centered at the youngest age within each age epoch.

† Sex-specific effects of PRS on BP slope were derived from the sex × PRS × age interaction term in a linear mixed model including centered age, sex, PRS, and their two-way interactions, and adjusted for age-adjusted height residual, age-adjusted BMI residual, and the first 10 genetic principal components. Age was centered at the youngest age within each age epoch.

Table S4. Sex-specific BP slope in three epochs

	Prepuberty		Puberty		Postpuberty	
	β (95% CI, mmHg)	*P*	β (95% CI, mmHg)	*P*	β (95% CI, mmHg)	*P*
**SBP**						
Continuous PRS						
Sex × age	0.08 (-0.20, 0.36)	0.570	-0.41 (-0.63, -0.18)	0.000	-0.54 (-0.78, -0.30)	1.10×10^{-5}
PRS quartile						
Sex × age	0.09 (-0.19, 0.36)	0.536	-0.42 (-0.64, -0.19)	0.000	-0.52 (-0.76, -0.28)	2.30×10^{-5}
**DBP**						
Continuous PRS						
Sex × age	-0.28 (-0.53, -0.04)	0.025	0.17 (0.03, 0.31)	0.017	-0.42 (-0.58, -0.25)	4.88×10^{-7}
PRS quartile						
Sex × age	-0.29 (-0.53, -0.04)	0.024	0.17 (0.03, 0.31)	0.020	-0.41 (-0.57, -0.25)	5.98×10^{-7}

CI, confidence interval; SBP, systolic blood pressure; DBP, diastolic blood pressure; PRS, polygenic risk score. Reference group is boy. Estimates were derived from the sex × age term in a linear mixed model including centered age, sex, PRS, age × PRS, and adjusted for age-adjusted height residual and age-adjusted BMI residual, and the first 10 genetic principal components. Age was centered around the youngest age in each age epoch.

Table S5. Comparison of results before and after including sex × age

	Without sex × age		With sex × age	
	β* (95% CI, mmHg)	*P*	β† (95% CI, mmHg)	*P*
**Prepuberty**				
**SBP**				
PRS	1.70 (1.12, 2.29)	1.32×10^{-8}	1.70 (1.12, 2.29)	1.30×10^{-8}
PRS × age	0.01 (-0.13, 0.15)	0.855	0.01 (-0.13, 0.15)	0.859
PRS_Q2	2.59 (0.95, 4.24)	0.002	2.60 (0.95, 4.24)	0.002
PRS_Q3	3.20 (1.56, 4.85)	0.0001	3.22 (1.57, 4.87)	0.000
PRS_Q4	4.49 (2.83, 6.15)	1.30×10^{-7}	4.49 (2.83, 6.15)	1.32×10^{-7}
PRS_Q2 × age	-0.18 (-0.57, 0.21)	0.360	-0.18 (-0.57, 0.21)	0.360
PRS_Q3 × age	-0.17 (-0.57, 0.22)	0.384	-0.18 (-0.57, 0.21)	0.372
PRS_Q4 × age	0.08 (-0.32, 0.47)	0.694	0.08 (-0.32, 0.47)	0.696
**DBP**				
PRS	1.68 (1.12, 2.24)	6.40×10^{-9}	2.08 (1.41, 2.75)	8.33×10^{-9}
PRS × age	-0.02 (-0.15, 0.10)	0.728	-0.02 (-0.14, 0.11)	0.778
PRS_Q2	1.18 (-0.39, 2.75)	0.140	1.11 (-0.46, 2.68)	0.165
PRS_Q3	2.49 (0.93, 4.05)	0.002	2.46 (0.90, 4.02)	0.002
PRS_Q4	3.98 (2.39, 5.58)	1.10×10^{-6}	3.92 (2.33, 5.51)	1.54×10^{-6}
PRS_Q2 × age	-0.04 (-0.39, 0.31)	0.816	-0.02 (-0.37, 0.33)	0.905
PRS_Q3 × age	-0.17 (-0.51, 0.18)	0.337	-0.16 (-0.50, 0.19)	0.371
PRS_Q4 × age	-0.03 (-0.38, 0.32)	0.860	-0.01 (-0.36, 0.33)	0.936
**Puberty**				
**SBP**				
PRS	1.65 (1.12, 2.18)	1.52×10^{-9}	1.65 (1.12, 2.18)	1.26×10^{-9}
PRS × age	0.11 (0.00, 0.22)	0.042	0.11 (0.00, 0.22)	0.042
PRS_Q2	1.70 (0.21, 3.20)	0.026	1.68 (0.19, 3.17)	0.027
PRS_Q3	2.93 (1.43, 4.43)	0.000	2.93 (1.44, 4.43)	0.000
PRS_Q4	4.21 (2.72, 5.71)	3.44×10^{-8}	4.21 (2.72, 5.70)	3.24×10^{-8}
PRS_Q2 × age	-0.04 (-0.34, 0.27)	0.807	-0.04 (-0.34, 0.27)	0.809
PRS_Q3 × age	0.02 (-0.29, 0.32)	0.905	0.02 (-0.28, 0.33)	0.881

PRS_Q4 × age	0.32 (0.02, 0.63)	0.038	0.32 (0.02, 0.63)	0.036
**DBP**				
PRS	0.85 (0.50, 1.19)	1.51×10^{-6}	0.85 (0.50, 1.19)	1.59×10^{-6}
PRS × age	0.07 (0.00, 0.13)	0.061	0.07 (0.00, 0.13)	0.060
PRS_Q2	1.16 (0.21, 2.12)	0.017	1.14 (0.18, 2.09)	0.020
PRS_Q3	1.48 (0.53, 2.44)	0.002	1.47 (0.52, 2.43)	0.003
PRS_Q4	2.36 (1.39, 3.34)	2.05×10^{-6}	2.36 (1.39, 3.33)	2.13×10^{-6}
PRS_Q2 × age	0.04 (-0.15, 0.23)	0.687	0.05 (-0.15, 0.24)	0.639
PRS_Q3 × age	0.06 (-0.13, 0.26)	0.524	0.07 (-0.13, 0.26)	0.505
PRS_Q4 × age	0.16 (-0.03, 0.36)	0.104	0.16 (-0.03, 0.36)	0.104
**Postpuberty**				
**SBP**				
PRS	2.92 (2.38, 3.46)	1.55×10^{-25}	2.86 (2.32, 3.40)	9.58×10^{-25}
PRS × age	-0.07 (-0.18, 0.04)	0.190	-0.06 (-0.17, 0.05)	0.261
PRS_Q2	2.87 (1.32, 4.42)	0.0003	2.81 (1.26, 4.36)	0.000
PRS_Q3	4.35 (2.76, 5.93)	8.10×10^{-8}	4.33 (2.75, 5.91)	8.45×10^{-8}
PRS_Q4	7.85 (6.28, 9.42)	2.36×10^{-22}	7.67 (6.10, 9.24)	1.77×10^{-21}
PRS_Q2 × age	-0.12 (-0.44, 0.20)	0.463	-0.11 (-0.43, 0.21)	0.503
PRS_Q3 × age	0.00 (-0.32, 0.33)	0.979	-0.01 (-0.33, 0.32)	0.973
PRS_Q4 × age	-0.24 (-0.55, 0.08)	0.147	-0.20 (-0.52, 0.12)	0.213
**DBP**				
PRS	1.18 (0.82, 1.55)	2.97×10^{-10}	1.14 (0.78, 1.51)	9.96×10^{-10}
PRS × age	0.08 (0.00, 0.15)	0.044	0.09 (0.01, 0.16)	0.023
PRS_Q2	1.25 (0.23, 2.28)	0.017	1.22 (0.20, 2.24)	0.019
PRS_Q3	1.60 (0.57, 2.63)	0.002	1.53 (0.51, 2.56)	0.003
PRS_Q4	2.84 (1.80, 3.87)	8.70×10^{-8}	2.75 (1.72, 3.78)	1.75×10^{-7}
PRS_Q2 × age	0.06 (-0.16, 0.28)	0.583	0.07 (-0.15, 0.28)	0.538
PRS_Q3 × age	0.12 (-0.09, 0.34)	0.270	0.14 (-0.08, 0.35)	0.219
PRS_Q4 × age	0.24 (0.02, 0.45)	0.033	0.25 (0.04, 0.47)	0.021

CI, confidence interval; SBP, systolic blood pressure; DBP, diastolic blood pressure; PRS, polygenic risk score.

* Estimates were derived from model which included age, sex, PRS, age × PRS, age-adjusted height residual, age-adjusted BMI residual and the first 10 genetic principal components. Age was centered around the youngest age in each age epoch.

† Estimates were derived from model which included age, sex, PRS, age × PRS, sex × age, age-adjusted height residual, age-adjusted BMI residual and the first 10 genetic principal components. Age was centered around the youngest age in each age epoch.

Table S6. Comparison of PRS effects on initial BP levels in three age epochs using LMM and GEE

	LMM		GEE	
	β* (95% CI, mmHg)	*P*	β (95% CI, mmHg)	*P*
**Prepuberty**				
SBP				
Continuous PRS	1.70 (1.12, 2.29)	1.32×10^{-8}	1.69 (1.11, 2.27)	1.24×10^{-8}
PRS quartile (ref=quartile 1)				
Quartile 2	2.59 (0.95, 4.24)	0.002	2.56 (0.91, 4.21)	0.002
Quartile 3	3.20 (1.56, 4.85)	0.0001	3.18 (1.42, 4.94)	0.0003
Quartile 4	4.49 (2.83, 6.15)	1.30×10^{-7}	4.50 (2.88, 6.11)	4.51×10^{-8}
DBP				
Continuous PRS	1.68 (1.12, 2.24)	6.40×10^{-9}	1.68 (1.08, 2.28)	3.80×10^{-8}
PRS quartile (ref=quartile 1)				
Quartile 2	1.18 (-0.39, 2.75)	0.140	1.18 (-0.38, 2.74)	0.140
Quartile 3	2.49 (0.93, 4.05)	0.002	2.52 (0.95, 4.09)	0.002
Quartile 4	3.98 (2.39, 5.58)	1.10×10^{-6}	3.98 (2.38, 5.57)	1.10×10^{-6}
**Puberty**				
SBP				
Continuous PRS	1.65 (1.12, 2.18)	1.52×10^{-9}	1.65 (1.12, 2.19)	1.51×10^{-9}
PRS quartile (ref=quartile 1)				
Quartile 2	1.70 (0.21, 3.20)	0.030	1.77 (0.30, 3.25)	0.019
Quartile 3	2.93 (1.43, 4.43)	0.0001	2.98 (1.48, 4.49)	0.0001
Quartile 4	4.21 (2.72, 5.71)	3.44×10^{-8}	4.24 (2.76, 5.73)	2.26×10^{-8}
DBP				
Continuous PRS	0.85 (0.50, 1.19)	1.51×10^{-6}	0.85 (0.49, 1.22)	4.17×10^{-6}
PRS quartile (ref=quartile 1)				
Quartile 2	1.16 (0.21, 2.12)	0.020	1.21 (0.29, 2.12)	0.010
Quartile 3	1.48 (0.53, 2.44)	0.002	1.50 (0.55, 2.45)	0.002
Quartile 4	2.36 (1.39, 3.34)	2.05×10^{-6}	2.38 (1.40, 3.35)	1.91×10^{-6}
**Postpuberty**				
SBP				

Continuous PRS	2.92 (2.38, 3.46)	1.55×10^{-25}	2.92 (2.40, 3.44)	0.000
PRS quartile (ref=quartile 1)				
Quartile 2	2.87 (1.32, 4.42)	0.0003	2.83 (1.33, 4.34)	0.0002
Quartile 3	4.35 (2.76, 5.93)	8.10×10^{-8}	4.32 (2.80, 5.84)	2.68×10^{-8}
Quartile 4	7.85 (6.28, 9.42)	2.36×10^{-22}	7.83 (6.35, 9.32)	0.000
DBP				
Continuous PRS	1.18 (0.82, 1.55)	2.97×10^{-10}	1.19 (0.82, 1.56)	3.31×10^{-10}
PRS quartile (ref=quartile 1)				
Quartile 2	1.25 (0.23, 2.28)	0.017	1.25 (0.27, 2.22)	0.012
Quartile 3	1.60 (0.57, 2.63)	0.002	1.55 (0.53, 2.57)	0.003
Quartile 4	2.84 (1.80, 3.87)	8.70×10^{-8}	2.84 (1.81, 3.88)	6.63×10^{-8}

PRS, polygenic risk score; BP, blood pressure; LMM, linear mixed model; GEE, generalized estimating equations; CI, confidence interval; DBP, diastolic blood pressure; ref, reference group; SBP, systolic blood pressure.

* For continuous PRS, effect size β is expressed in mmHg of initial BP levels per 1 standard deviation (SD) in the PRS; for PRS quartiles, effect size β is reported as mean difference of initial BP levels compared with the lowest quartile.

Table S7. Comparison of PRS effects on BP slope in three age epochs using LMM and GEE

	LMM		GEE	
	β* (95% CI, mmHg)	*P*	β (95% CI, mmHg)	*P*
**Prepuberty**				
SBP				
PRS × age	0.01 (-0.13, 0.15)	0.855	0.01 (-0.12, 0.15)	0.859
PRS quartile (ref=Q1)				
PRS_Q2 × age	-0.18 (-0.57, 0.21)	0.360	-0.18 (-0.56, 0.20)	0.358
PRS_Q3 × age	-0.17 (-0.57, 0.22)	0.384	-0.17 (-0.59, 0.25)	0.424
PRS_Q4 × age	0.08 (-0.32, 0.47)	0.694	0.07 (-0.32, 0.46)	0.717
DBP				
PRS × age	-0.02 (-0.15, 0.10)	0.728	-0.02 (-0.15, 0.11)	0.734
PRS quartile (ref=Q1)				
PRS_Q2 × age	-0.04 (-0.39, 0.31)	0.816	-0.04 (-0.38, 0.30)	0.813
PRS_Q3 × age	-0.17 (-0.51, 0.18)	0.337	-0.18 (-0.53, 0.17)	0.310
PRS_Q4 × age	-0.03 (-0.38, 0.32)	0.860	-0.03 (-0.38, 0.32)	0.865
**Puberty**				
SBP				
PRS × age	0.11 (0.00, 0.22)	0.042	0.11 (0.00, 0.22)	0.052
PRS quartile (ref=Q1)				
PRS_Q2 × age	-0.04 (-0.34, 0.27)	0.807	-0.05 (-0.36, 0.25)	0.724
PRS_Q3 × age	0.02 (-0.29, 0.32)	0.905	0.00 (-0.30, 0.31)	0.982
PRS_Q4 × age	0.32 (0.02, 0.63)	0.038	0.31 (0.00, 0.61)	0.047
DBP				
PRS × age	0.07 (0.00, 0.13)	0.061	0.07 (-0.01, 0.14)	0.073
PRS quartile (ref=Q1)				
PRS_Q2 × age	0.04 (-0.15, 0.23)	0.687	0.03 (-0.16, 0.21)	0.772
PRS_Q3 × age	0.06 (-0.13, 0.26)	0.524	0.06 (-0.13, 0.26)	0.528
PRS_Q4 × age	0.16 (-0.03, 0.36)	0.104	0.16 (-0.04, 0.36)	0.112
**Postpuberty**				
SBP				

PRS × age	-0.07 (-0.18, 0.04)	0.190	-0.07 (-0.18, 0.03)	0.181
PRS quartile (ref=Q1)				
PRS_Q2 × age	-0.12 (-0.44, 0.20)	0.463	-0.11 (-0.43, 0.21)	0.498
PRS_Q3 × age	0.00 (-0.32, 0.33)	0.979	0.01 (-0.31, 0.33)	0.939
PRS_Q4 × age	-0.24 (-0.55, 0.08)	0.147	-0.23 (-0.53, 0.08)	0.144
DBP				
PRS × age	0.08 (0.00, 0.15)	0.044	0.08 (0.00, 0.15)	0.046
PRS quartile (ref=Q1)				
PRS_Q2 × age	0.06 (-0.16, 0.28)	0.583	0.06 (-0.14, 0.27)	0.535
PRS_Q3 × age	0.12 (-0.09, 0.34)	0.270	0.14 (-0.07, 0.35)	0.192
PRS_Q4 × age	0.24 (0.02, 0.45)	0.033	0.23 (0.02, 0.44)	0.030

PRS, polygenic risk score; BP, blood pressure; LMM, linear mixed model; GEE, generalized estimating equations; CI, confidence interval; DBP, diastolic blood pressure; ref, reference group; SBP, systolic blood pressure.

*For continuous PRS, effect size β is expressed in mmHg/year rate of change of BP per 1 standard deviation (SD) in the PRS; for PRS quartiles, effect size β is reported as mean difference of BP rates of change compared with the lowest quartile.

Table S8. PRS effects on BP trajectories in the three age epochs using height and BMI instead of their age-adjusted residuals

	PRS effect on initial BP levels			PRS effect on BP slopes	
	β* (95% CI, mmHg)	*P*		β† (95% CI, mmHg)	*P*
**Prepuberty**					
**SBP**					
Continuous PRS	1.70 (1.12, 2.29)	1.32×10^{-8}		0.01 (-0.13, 0.15)	0.855
PRS quartile (ref=quartile 1)					
Quartile 2	2.59 (0.95, 4.24)	0.002		-0.18 (-0.57, 0.21)	0.360
Quartile 3	3.20 (1.56, 4.85)	0.0001		-0.17 (-0.57, 0.22)	0.384
Quartile 4	4.49 (2.83, 6.15)	1.30×10^{-7}		0.08 (-0.32, 0.47)	0.694
**DBP**					
Continuous PRS	1.68 (1.12, 2.24)	6.40×10^{-9}		-0.02 (-0.15, 0.10)	0.728
PRS quartile (ref=quartile 1)					
Quartile 2	1.18 (-0.39, 2.75)	0.140		-0.04 (-0.39, 0.31)	0.816
Quartile 3	2.49 (0.93, 4.05)	0.002		-0.17 (-0.51, 0.18)	0.337
Quartile 4	3.98 (2.39, 5.58)	1.10×10^{-6}		-0.03 (-0.38, 0.32)	0.860
**Puberty**					
**SBP**					
Continuous PRS	1.65 (1.12, 2.18)	1.52×10^{-9}		0.11 (0.00, 0.22)	0.042
PRS quartile (ref=quartile 1)					
Quartile 2	1.70 (0.21, 3.20)	0.026		-0.04 (-0.34, 0.27)	0.807
Quartile 3	2.93 (1.43, 4.43)	0.0001		0.02 (-0.29, 0.32)	0.905
Quartile 4	4.21 (2.72, 5.71)	3.44×10^{-8}		0.32 (0.02, 0.63)	0.038
**DBP**					
Continuous PRS	0.85 (0.50, 1.19)	1.51×10^{-6}		0.07 (0.00, 0.13)	0.061
PRS quartile (ref=quartile 1)					
Quartile 2	1.16 (0.21, 2.12)	0.017		0.04 (-0.15, 0.23)	0.687
Quartile 3	1.48 (0.53, 2.44)	0.002		0.06 (-0.13, 0.26)	0.524
Quartile 4	2.36 (1.39, 3.34)	2.05×10^{-6}		0.16 (-0.03, 0.36)	0.104
**Postpuberty**					
**SBP**					
Continuous PRS	2.92 (2.38, 3.46)	1.55×10^{-25}		-0.07 (-0.18, 0.04)	0.190
PRS quartile (ref=quartile 1)					
Quartile 2	2.87 (1.32, 4.42)	0.0003		-0.12 (-0.44, 0.20)	0.463
Quartile 3	4.35 (2.76, 5.93)	8.10×10^{-8}		0.00 (-0.32, 0.33)	0.979
Quartile 4	7.85 (6.28, 9.42)	2.36×10^{-22}		-0.24 (-0.55, 0.08)	0.147
**DBP**					
Continuous PRS	1.18 (0.82, 1.55)	2.97×10^{-10}		0.08 (0.00, 0.15)	0.044
PRS quartile (ref=quartile 1)					
Quartile 2	1.25 (0.23, 2.28)	0.017		0.06 (-0.16, 0.28)	0.583

Quartile 3	1.60 (0.57, 2.63)	0.002		0.12 (-0.09, 0.34)	0.270
Quartile 4	2.84 (1.80, 3.87)	8.70×10^{-8}		0.24 (0.02, 0.45)	0.033

CI, confidence interval; DBP, diastolic blood pressure; PRS, polygenic risk score; ref, reference group; SBP, systolic blood pressure. For each time frame, results are extracted from linear mixed models with adjustment for sex, height, BMI and the first 10 genetic principal components. Age was centered around the youngest age in each age epoch.

*For continuous PRS, effect size β is expressed in mmHg of initial BP levels per 1 standard deviation (SD) in the PRS; for PRS quartiles, effect size β is reported as mean difference of initial BP levels compared with the lowest quartile.

†For continuous PRS, effect size β is expressed in mmHg/year rate of change of BP per 1 SD in the PRS; for PRS quartiles, effect size β is reported as mean difference of BP rates of change compared with the lowest quartile.

Table S9. PA effects on DBP trajectories in the three age epochs

	PA effect on initial DBP levels			PA effect on rates of DBP changes	
	β* (95% CI, mmHg)	*P*		β† (95% CI, mmHg)	*P*
**Prepuberty**					
Model 1					
PA	0.18 (-1.58, 1.94)	0.841		0.09 (-0.27, 0.46)	0.616
Model 2					
PA	0.13 (-1.61, 1.86)	0.886		0.09 (-0.27, 0.46)	0.615
Model 3					
PA	0.22 (-1.52, 1.96)	0.803		0.07 (-0.30, 0.44)	0.715
PRS	1.57 (0.37, 2.76)	0.011		-0.07 (-0.32, 0.17)	0.556
PA*PRS	0.50 (-1.18, 2.19)	0.558		-0.09 (-0.44, 0.27)	0.638
**Puberty**					
Model 1					
PA	-0.79 (-1.93, 0.36)	0.178		0.17 (-0.18, 0.53)	0.340
Model 2					
PA	-0.76 (-1.91, 0.38)	0.191		0.16 (-0.20, 0.51)	0.383
Model 3					
PA	-0.82 (-1.96, 0.33)	0.162		0.18 (-0.18, 0.53)	0.333
PRS	0.65 (-0.16, 1.45)	0.117		0.23 (-0.01, 0.48)	0.060
PA*PRS	0.36 (-0.80, 1.52)	0.542		0.02 (-0.34, 0.37)	0.928
**Postpuberty**					
Model 1					
PA	-0.71 (-1.71, 0.29)	0.162		0.18 (-0.18, 0.54)	0.320
Model 2					
PA	-0.72 (-1.72, 0.28)	0.158		0.18 (-0.18, 0.53)	0.333
Model 3					
PA	-0.75 (-1.75, 0.26)	0.145		0.18 (-0.17, 0.54)	0.311
PRS	0.68 (-0.04, 1.40)	0.065		0.13 (-0.12, 0.37)	0.314
PA*PRS	0.81 (-0.24, 1.85)	0.130		-0.17 (-0.53, 0.20)	0.375

DBP indicates diastolic blood pressure; PA, physical activity; PRS, polygenic risk score. Analyses were conducted in the subsets of individuals who had PA data (N=488 in prepuberty, 696 in puberty, and 874 in postpuberty). Model 1 included centered age, PA, PA × age, and adjusted for the PRS, sex, height residual regressed from age, and the first genetic 10 PCs (season of PA measurement and mean wear time were also adjusted in GECKO Drenthe). Model 2 further adjusted BMI residual regressed from age. Model 3 added the interaction of PA and the PRS. Age was centered around the youngest age in each age epoch (age 5 years for prepuberty, 12 years for puberty, 15 years for postpuberty).

*For PA, effect size β represents the difference in initial DBP level (mm Hg) between the active and reference groups; for PRS, β represents the difference in initial DBP level (mm Hg) per 1 SD increase in PRS.

†For PA, effect size β represents the difference in the annual rate of DBP change (mm Hg/y) between the active and reference groups; for PRS, β represents the difference in the annual rate of DBP change (mm Hg/y) per 1 SD increase in PRS.

Table S10. Comparison of PA effects on initial BP levels in three age epochs using LMM and GEE

	LMM		GEE	
	β (95% CI, mmHg)	*P*	β (95% CI, mmHg)	*P*
**Prepuberty**				
SBP				
Model 1 PA	0.21 (-1.63, 2.06)	0.822	0.27 (-1.56, 2.10)	0.774
Model 2 PA	0.14 (-1.67, 1.96)	0.878	0.20 (-1.59, 1.99)	0.829
Model 3 PA	0.24 (-1.58, 2.06)	0.793	0.30 (-1.47, 2.08)	0.738
Model 3 PRS	1.19 (-0.04, 2.43)	0.059	1.19 (0.12, 2.27)	0.030
Model 3 PA × PRS	1.19 (-0.57, 2.95)	0.186	1.23 (-0.45, 2.91)	0.151
DBP				
Model 1 PA	0.18 (-1.58, 1.94)	0.841	0.19 (-1.55, 1.94)	0.828
Model 2 PA	0.13 (-1.61, 1.86)	0.886	0.13 (-1.59, 1.85)	0.879
Model 3 PA	0.22 (-1.52, 1.96)	0.803	0.22 (-1.49, 1.93)	0.801
Model 3 PRS	1.57 (0.37, 2.76)	0.011	1.56 (0.55, 2.58)	0.003
Model 3 PA × PRS	0.50 (-1.18, 2.19)	0.558	0.50 (-1.06, 2.06)	0.529
**Puberty**				
SBP				
Model 1 PA	-1.90 (-3.77, -0.03)	0.046	-1.96 (-3.80, -0.13)	0.036
Model 2 PA	-1.78 (-3.62, 0.07)	0.060	-1.83 (-3.63, -0.02)	0.048
Model 3 PA	-1.82 (-3.68, 0.03)	0.055	-1.88 (-3.69, -0.07)	0.042
Model 3 PRS	1.36 (0.09, 2.63)	0.036	1.38 (0.13, 2.63)	0.031
Model 3 PA × PRS	0.99 (-0.89, 2.87)	0.301	0.97 (-0.74, 2.67)	0.267
DBP				
Model 1 PA	-0.79 (-1.93, 0.36)	0.178	-0.78 (-1.92, 0.35)	0.176
Model 2 PA	-0.76 (-1.91, 0.38)	0.191	-0.76 (-1.90, 0.38)	0.191
Model 3 PA	-0.82 (-1.96, 0.33)	0.162	-0.82 (-1.95, 0.31)	0.153
Model 3 PRS	0.65 (-0.16, 1.45)	0.117	0.64 (-0.17, 1.45)	0.119
Model 3 PA × PRS	0.36 (-0.80, 1.52)	0.542	0.43 (-0.73, 1.59)	0.467
**Postpuberty**				
SBP				

Model 1 PA	-0.35 (-1.87, 1.16)	0.649	-0.33 (-1.81, 1.15)	0.659
Model 2 PA	-0.41 (-1.91, 1.09)	0.589	-0.42 (-1.87, 1.04)	0.573
Model 3 PA	-0.42 (-1.92, 1.09)	0.588	-0.42 (-1.87, 1.03)	0.573
Model 3 PRS	2.84 (1.82, 3.86)	6.89×10^{-8}	2.85 (1.91, 3.79)	2.69×10^{-9}
Model 3 PA × PRS	0.21 (-1.30, 1.71)	0.788	0.18 (-1.21, 1.57)	0.799
DBP				
Model 1 PA	-0.71 (-1.71, 0.29)	0.162	-0.71 (-1.71, 0.28)	0.161
Model 2 PA	-0.72 (-1.72, 0.28)	0.158	-0.73 (-1.73, 0.27)	0.153
Model 3 PA	-0.75 (-1.75, 0.26)	0.145	-0.75 (-1.75, 0.24)	0.138
Model 3 PRS	0.68 (-0.04, 1.40)	0.065	0.69 (-0.02, 1.40)	0.056
Model 3 PA × PRS	0.81 (-0.24, 1.85)	0.130	0.79 (-0.29, 1.87)	0.150

PA, physical activity; BP, blood pressure; LMM, linear mixed model; GEE, generalized estimating equations; CI, confidence interval; PRS, polygenic risk score; DBP, diastolic blood pressure; SBP, systolic blood pressure. Model 1 included centered age, PA, PA × age, and adjusted for the PRS, sex, height residual regressed from age, and the first genetic 10 PCs (season of PA measurement and mean wear time were also adjusted in GECKO Drenthe). Model 2 further adjusted BMI residual regressed from age. Model 3 added the interaction of PA and the PRS. Age was centered around the youngest age in each age epoch.

Table S11. Comparison of PA effects on BP slope in three age epochs using LMM and GEE

	LMM		GEE	
	β (95% CI, mmHg)	*P*	β (95% CI, mmHg)	*P*
**Prepuberty**				
SBP				
Model 1 PA × age	0.07 (-0.34, 0.49)	0.735	0.06 (-0.36, 0.47)	0.792
Model 2 PA × age	0.07 (-0.35, 0.48)	0.753	0.05 (-0.36, 0.47)	0.801
Model 3 PA × age	0.04 (-0.38, 0.45)	0.854	0.02 (-0.39, 0.44)	0.908
Model 3 PRS × age	0.06 (-0.23, 0.34)	0.701	0.05 (-0.21, 0.31)	0.685
Model 3 PA × PRS × age	-0.26 (-0.67, 0.16)	0.227	-0.28 (-0.66, 0.11)	0.165
DBP				
Model 1 PA × age	0.09 (-0.27, 0.46)	0.616	0.10 (-0.27, 0.47)	0.598
Model 2 PA × age	0.09 (-0.27, 0.46)	0.615	0.10 (-0.27, 0.47)	0.592
Model 3 PA × age	0.07 (-0.30, 0.44)	0.715	0.07 (-0.29, 0.44)	0.690
Model 3 PRS × age	-0.07 (-0.32, 0.17)	0.556	-0.07 (-0.29, 0.15)	0.522
Model 3 PA × PRS × age	-0.09 (-0.44, 0.27)	0.638	-0.09 (-0.43, 0.24)	0.591
**Puberty**				
SBP				
Model 1 PA × age	0.16 (-0.43, 0.75)	0.588	0.20 (-0.38, 0.78)	0.505
Model 2 PA × age	0.08 (-0.50, 0.66)	0.790	0.10 (-0.47, 0.67)	0.725
Model 3 PA × age	0.10 (-0.48, 0.68)	0.735	0.13 (-0.44, 0.69)	0.665
Model 3 PRS × age	0.34 (-0.04, 0.73)	0.081	0.34 (-0.05, 0.72)	0.084
Model 3 PA × PRS × age	-0.50 (-1.07, 0.08)	0.091	-0.50 (-1.01, 0.02)	0.058
DBP				
Model 1 PA × age	0.17 (-0.18, 0.53)	0.339	0.18 (-0.18, 0.54)	0.332
Model 2 PA × age	0.16 (-0.20, 0.51)	0.382	0.16 (-0.20, 0.52)	0.382
Model 3 PA × age	0.18 (-0.18, 0.53)	0.333	0.18 (-0.17, 0.54)	0.317
Model 3 PRS × age	0.24 (-0.01, 0.48)	0.060	0.24 (0.00, 0.48)	0.054
Model 3 PA × PRS × age	0.02 (-0.34, 0.37)	0.928	-0.02 (-0.39, 0.34)	0.909
**Postpuberty**				
SBP				

Model 1 PA × age	0.37 (-0.17, 0.92)	0.182	0.35 (-0.18, 0.87)	0.193
Model 2 PA × age	0.33 (-0.18, 0.85)	0.203	0.33 (-0.17, 0.83)	0.197
Model 3 PA × age	0.33 (-0.18, 0.85)	0.207	0.33 (-0.17, 0.83)	0.200
Model 3 PRS × age	-0.08 (-0.42, 0.25)	0.629	-0.09 (-0.39, 0.21)	0.555
Model 3 PA × PRS × age	-0.02 (-0.54, 0.50)	0.944	0.00 (-0.51, 0.52)	0.994
DBP				
Model 1 PA × age	0.18 (-0.18, 0.54)	0.320	0.17 (-0.18, 0.53)	0.335
Model 2 PA × age	0.18 (-0.18, 0.53)	0.333	0.17 (-0.18, 0.52)	0.336
Model 3 PA × age	0.18 (-0.17, 0.54)	0.311	0.18 (-0.17, 0.53)	0.312
Model 3 PRS × age	0.13 (-0.12, 0.37)	0.314	0.12 (-0.14, 0.38)	0.352
Model 3 PA × PRS × age	-0.17 (-0.53, 0.20)	0.375	-0.16 (-0.56, 0.24)	0.428

PA, physical activity; BP, blood pressure; LMM, linear mixed model; GEE, generalized estimating equations; CI, confidence interval; PRS, polygenic risk score; DBP, diastolic blood pressure; SBP, systolic blood pressure. Model 1 included centered age, PA, PA × age, and adjusted for the PRS, sex, height residual regressed from age and the first genetic 10 PCs (season of PA measurement and mean wear time were also adjusted in GECKO Drenthe). Model 2 further adjusted BMI residual regressed from age. Model 3 added the interaction of PA and the PRS. Age was centered around the youngest age in each age epoch.

Table S12. PA effects on SBP trajectories in the three age epochs using height and BMI instead of their age-adjusted residuals

	PA effect on initial SBP levels			PA effect on rates of SBP changes	
	β* (95% CI, mmHg)	*P*		β† (95% CI, mmHg)	*P*
**Prepuberty**					
Model 1					
PA	0.21 (-1.63, 2.06)	0.822		0.07 (-0.34, 0.49)	0.735
Model 2					
PA	0.14 (-1.67, 1.96)	0.878		0.07 (-0.35, 0.48)	0.753
Model 3					
PA	0.24 (-1.58, 2.06)	0.793		0.04 (-0.38, 0.45)	0.854
PRS	1.19 (-0.04, 2.43)	0.059		0.06 (-0.23, 0.34)	0.701
PA*PRS	1.19 (-0.57, 2.95)	0.186		-0.26 (-0.67, 0.16)	0.227
**Puberty**					
Model 1					
PA	-1.90 (-3.77, -0.03)	0.046		0.16 (-0.43, 0.75)	0.588
Model 2					
PA	-1.78 (-3.62, 0.07)	0.060		0.08 (-0.50, 0.66)	0.790
Model 3					
PA	-1.82 (-3.68, 0.03)	0.055		0.10 (-0.48, 0.68)	0.738
PRS	1.36 (0.09, 2.63)	0.036		0.35 (-0.04, 0.73)	0.081
PA*PRS	0.99 (-0.89, 2.87)	0.301		-0.50 (-1.08, 0.08)	0.092
**Postpuberty**					
Model 1					
PA	-0.35 (-1.87, 1.16)	0.649		0.37 (-0.17, 0.92)	0.182
Model 2					
PA	-0.41 (-1.91, 1.09)	0.589		0.33 (-0.18, 0.85)	0.203
Model 3					
PA	-0.42 (-1.92, 1.09)	0.588		0.33 (-0.18, 0.85)	0.207
PRS	2.84 (1.82, 3.86)	6.89×10^{-8}		-0.08 (-0.42, 0.25)	0.629
PA*PRS	0.21 (-1.30, 1.71)	0.788		-0.02 (-0.54, 0.5)	0.944

CI, confidence interval; PA, physical activity; PRS, polygenic risk score; SBP, systolic blood pressure. Analyses were conducted in the subsets of individuals who had PA data (N=488 in prepuberty, 696 in puberty, and 874 in postpuberty). Model 1 included centered age, PA, PA × age, and adjusted for the PRS, sex, height and the first genetic 10 PCs (season of PA measurement and mean wear time were also adjusted in GECKO Drenthe). Model 2 further adjusted BMI. Model 3 added the interaction of PA and the PRS. Age was centered around the youngest age in each age epoch.

Table S13. PA effects on DBP trajectories in the three age epochs using height and BMI instead of their age-adjusted residuals

	PA effect on initial DBP levels			PA effect on rates of DBP changes	
	β* (95% CI, mmHg)	*P*		β† (95% CI, mmHg)	*P*
**Prepuberty**					
Model 1					
PA	0.18 (-1.58, 1.94)	0.841		0.09 (-0.27, 0.46)	0.616
Model 2					
PA	0.13 (-1.61, 1.86)	0.886		0.09 (-0.27, 0.46)	0.615
Model 3					
PA	0.22 (-1.52, 1.96)	0.803		0.07 (-0.30, 0.44)	0.715
PRS	1.57 (0.37, 2.76)	0.011		-0.07 (-0.32, 0.17)	0.556
PA*PRS	0.50 (-1.18, 2.19)	0.558		-0.09 (-0.44, 0.27)	0.638
**Puberty**					
Model 1					
PA	-0.79 (-1.93, 0.36)	0.178		0.17 (-0.18, 0.53)	0.340
Model 2					
PA	-0.76 (-1.91, 0.38)	0.191		0.16 (-0.20, 0.51)	0.383
Model 3					
PA	-0.82 (-1.96, 0.33)	0.162		0.18 (-0.18, 0.53)	0.333
PRS	0.65 (-0.16, 1.45)	0.117		0.23 (-0.01, 0.48)	0.060
PA*PRS	0.36 (-0.80, 1.52)	0.542		0.02 (-0.34, 0.37)	0.928
**Postpuberty**					
Model 1					
PA	-0.71 (-1.71, 0.29)	0.162		0.18 (-0.18, 0.54)	0.320
Model 2					
PA	-0.72 (-1.72, 0.28)	0.158		0.18 (-0.18, 0.53)	0.333
Model 3					
PA	-0.75 (-1.75, 0.26)	0.145		0.18 (-0.17, 0.54)	0.311
PRS	0.68 (-0.04, 1.40)	0.065		0.13 (-0.12, 0.37)	0.314
PA*PRS	0.81 (-0.24, 1.85)	0.130		-0.17 (-0.53, 0.20)	0.375

DBP indicates diastolic blood pressure; PA, physical activity; PRS, polygenic risk score. Analyses were conducted in the subsets of individuals who had PA data (N=488 in prepuberty, 696 in puberty, and 874 in postpuberty). Model 1 included centered age, PA, PA × age, and adjusted for the PRS, sex, height and the first genetic 10 PCs (season of PA measurement and mean wear time were also adjusted in GECKO Drenthe). Model 2 further adjusted BMI. Model 3 added the interaction of PA and the PRS. Age was centered around the youngest age in each age epoch.

Table S14. Comparison of PA effect on initial BP levels in three age epochs using different PA categories

	Dichotomized PA		Continuous/Ordinal PA	
	β (95% CI, mmHg)	*P*	β (95% CI, mmHg)	*P*
**Prepuberty**				
SBP				
Model 1 PA	0.21 (-1.63, 2.06)	0.822	0.01 (-0.02, 0.05)	0.466
Model 2 PA	0.14 (-1.67, 1.96)	0.878	0.01 (-0.03, 0.05)	0.545
Model 3 PA	0.24 (-1.58, 2.06)	0.793	0.01 (-0.03, 0.05)	0.527
Model 3 PRS	1.19 (-0.04, 2.43)	0.059	1.80 (0.92, 2.68)	7.20×10^{-5}
Model 3 PA × PRS	1.19 (-0.57, 2.95)	0.186	0.02 (-0.02, 0.05)	0.404
DBP				
Model 1 PA	0.18 (-1.58, 1.94)	0.841	0.01 (-0.02, 0.05)	0.480
Model 2 PA	0.13 (-1.61, 1.86)	0.886	0.01 (-0.02, 0.05)	0.545
Model 3 PA	0.22 (-1.52, 1.96)	0.803	0.01 (-0.02, 0.05)	0.492
Model 3 PRS	1.57 (0.37, 2.76)	0.011	1.84 (1.00, 2.69)	2.09×10^{-5}
Model 3 PA × PRS	0.50 (-1.18, 2.19)	0.558	0.02 (-0.02, 0.05)	0.275
**Puberty**				
SBP				
Model 1 PA	-1.90 (-3.77, -0.03)	0.046	-0.7 (-1.20, -0.21)	0.006
Model 2 PA	-1.78 (-3.62, 0.07)	0.060	-0.63 (-1.12, -0.14)	0.011
Model 3 PA	-1.82 (-3.68, 0.03)	0.055	-0.66 (-1.15, -0.17)	0.009
Model 3 PRS	1.36 (0.09, 2.63)	0.036	1.79 (0.85, 2.72)	0.000
Model 3 PA × PRS	0.99 (-0.89, 2.87)	0.301	-0.05(-0.56, 0.46)	0.850
DBP				
Model 1 PA	-0.79 (-1.93, 0.36)	0.178	-0.27 (-0.57, 0.03)	0.083
Model 2 PA	-0.76 (-1.91, 0.38)	0.191	-0.26 (-0.56, 0.05)	0.098
Model 3 PA	-0.82 (-1.96, 0.33)	0.162	-0.26 (-0.57, 0.04)	0.091
Model 3 PRS	0.65 (-0.16, 1.45)	0.117	0.82 (0.24, 1.40)	0.006
Model 3 PA × PRS	0.36 (-0.80, 1.52)	0.542	-0.09 (-0.41, 0.22)	0.567
**Postpuberty**				

SBP				
Model 1 PA	-0.35 (-1.87, 1.16)	0.649	-0.12 (-0.49, 0.25)	0.537
Model 2 PA	-0.41 (-1.91, 1.09)	0.589	-0.16 (-0.52, 0.21)	0.404
Model 3 PA	-0.42 (-1.92, 1.09)	0.588	-0.15 (-0.52, 0.22)	0.422
Model 3 PRS	2.84 (1.82, 3.86)	6.89×10^{-8}	2.92 (2.17, 3.67)	7.14×10^{-14}
Model 3 PA × PRS	0.21 (-1.30, 1.71)	0.788	-0.02 (-0.37, 0.33)	0.908
DBP				
Model 1 PA	-0.71 (-1.71, 0.29)	0.162	-0.08 (-0.32, 0.17)	0.549
Model 2 PA	-0.72 (-1.72, 0.28)	0.158	-0.08 (-0.33, 0.16)	0.515
Model 3 PA	-0.75 (-1.75, 0.26)	0.145	-0.10 (-0.35, 0.14)	0.409
Model 3 PRS	0.68 (-0.04, 1.40)	0.065	1.08 (0.56, 1.60)	5.58×10^{-5}
Model 3 PA × PRS	0.81 (-0.24, 1.85)	0.130	0.23 (-0.02, 0.48)	0.068

PA, physical activity; BP, blood pressure; CI, confidence interval; PRS, polygenic risk score; DBP, diastolic blood pressure; SBP, systolic blood pressure. Model 1 included centered age, PA, PA × age, and adjusted for the PRS, sex, height residual regressed from age and the first genetic 10 PCs (season of PA measurement and mean wear time were also adjusted in GECKO Drenthe). Model 2 further adjusted BMI residual regressed from age. Model 3 added the interaction of PA and the PRS. Age was centered around the youngest age in each age epoch.

Table S15. Comparison of PA effect on BP slope in three age epochs using different PA categories

	Dichotomized PA		Continuous/Ordinal PA	
	β (95% CI, mmHg)	*P*	β (95% CI, mmHg)	*P*
**Prepuberty**				
SBP				
Model 1 PA × age	0.07 (-0.34, 0.49)	0.735	0.00 (-0.01, 0.01)	0.473
Model 2 PA × age	0.07 (-0.35, 0.48)	0.753	0.00 (-0.01, 0.00)	0.417
Model 3 PA × age	0.04 (-0.38, 0.45)	0.854	0.00 (-0.01, 0.00)	0.393
Model 3 PRS × age	0.06 (-0.23, 0.34)	0.701	-0.08 (-0.28, 0.13)	0.463
Model 3 PA× PRS × age	-0.26 (-0.67, 0.16)	0.227	0.00 (-0.01, 0.01)	0.508
DBP				
Model 1 PA × age	0.09 (-0.27, 0.46)	0.616	0.00 (-0.01, 0.01)	0.728
Model 2 PA × age	0.09 (-0.27, 0.46)	0.615	0.00 (-0.01, 0.01)	0.681
Model 3 PA × age	0.07 (-0.30, 0.44)	0.715	0.00 (-0.01, 0.01)	0.633
Model 3 PRS × age	-0.07 (-0.32, 0.17)	0.556	-0.13 (-0.31, 0.05)	0.154
Model 3 PA × PRS × age	-0.09 (-0.44, 0.27)	0.638	0.00 (-0.01, 0.00)	0.199
**Puberty**				
SBP				
Model 1 PA × age	0.16 (-0.43, 0.75)	0.588	0.10 (-0.06, 0.25)	0.224
Model 2 PA × age	0.08 (-0.50, 0.66)	0.790	0.07 (-0.08, 0.23)	0.345
Model 3 PA × age	0.10 (-0.48, 0.68)	0.735	0.09 (-0.06, 0.24)	0.256
Model 3 PRS × age	0.34 (-0.04, 0.73)	0.081	0.11 (-0.17, 0.40)	0.437
Model 3 PA × PRS × age	-0.50 (-1.07, 0.08)	0.091	-0.07 (-0.22, 0.08)	0.352
DBP				
Model 1 PA × age	0.17 (-0.18, 0.53)	0.339	0.03 (-0.06, 0.13)	0.503
Model 2 PA × age	0.16 (-0.2, 0.51)	0.382	0.03 (-0.07, 0.12)	0.558
Model 3 PA × age	0.18 (-0.18, 0.53)	0.333	0.04 (-0.06, 0.13)	0.448
Model 3 PRS × age	0.24 (-0.01, 0.48)	0.060	0.24 (0.06, 0.42)	0.008
Model 3 PA × PRS × age	0.02 (-0.34, 0.37)	0.928	0.00 (-0.10, 0.09)	0.950
**Postpuberty**				
SBP				

Model 1 PA × age	0.37 (-0.17, 0.92)	0.182	0.09 (-0.04, 0.23)	0.161
Model 2 PA × age	0.33 (-0.18, 0.85)	0.203	0.08 (-0.04, 0.20)	0.200
Model 3 PA × age	0.33 (-0.18, 0.85)	0.207	0.08 (-0.05, 0.20)	0.215
Model 3 PRS × age	-0.08 (-0.42, 0.25)	0.629	-0.09 (-0.35, 0.17)	0.515
Model 3 PA × PRS × age	-0.02 (-0.54, 0.50)	0.944	0.01 (-0.11, 0.13)	0.871
DBP				
Model 1 PA × age	0.18 (-0.18, 0.54)	0.320	0.02 (-0.07, 0.10)	0.710
Model 2 PA × age	0.18 (-0.18, 0.53)	0.333	0.01 (-0.07, 0.10)	0.738
Model 3 PA × age	0.18 (-0.17, 0.54)	0.311	0.02 (-0.06, 0.11)	0.605
Model 3 PRS × age	0.13 (-0.12, 0.37)	0.314	0.03 (-0.15, 0.21)	0.762
Model 3 PA × PRS × age	-0.17 (-0.53, 0.20)	0.375	-0.12 (-0.21, -0.04)	0.006

PA, physical activity; BP, blood pressure; CI, confidence interval; PRS, polygenic risk score; DBP, diastolic blood pressure; SBP, systolic blood pressure. Model 1 included centered age, PA, PA × age, and adjusted for the PRS, sex, height residual regressed from age and the first genetic 10 PCs (season of PA measurement and mean wear time were also adjusted in GECKO Drenthe). Model 2 further adjusted BMI residual regressed from age. Model 3 added the interaction of PA and the PRS. Age was centered around the youngest age in each age epoch.

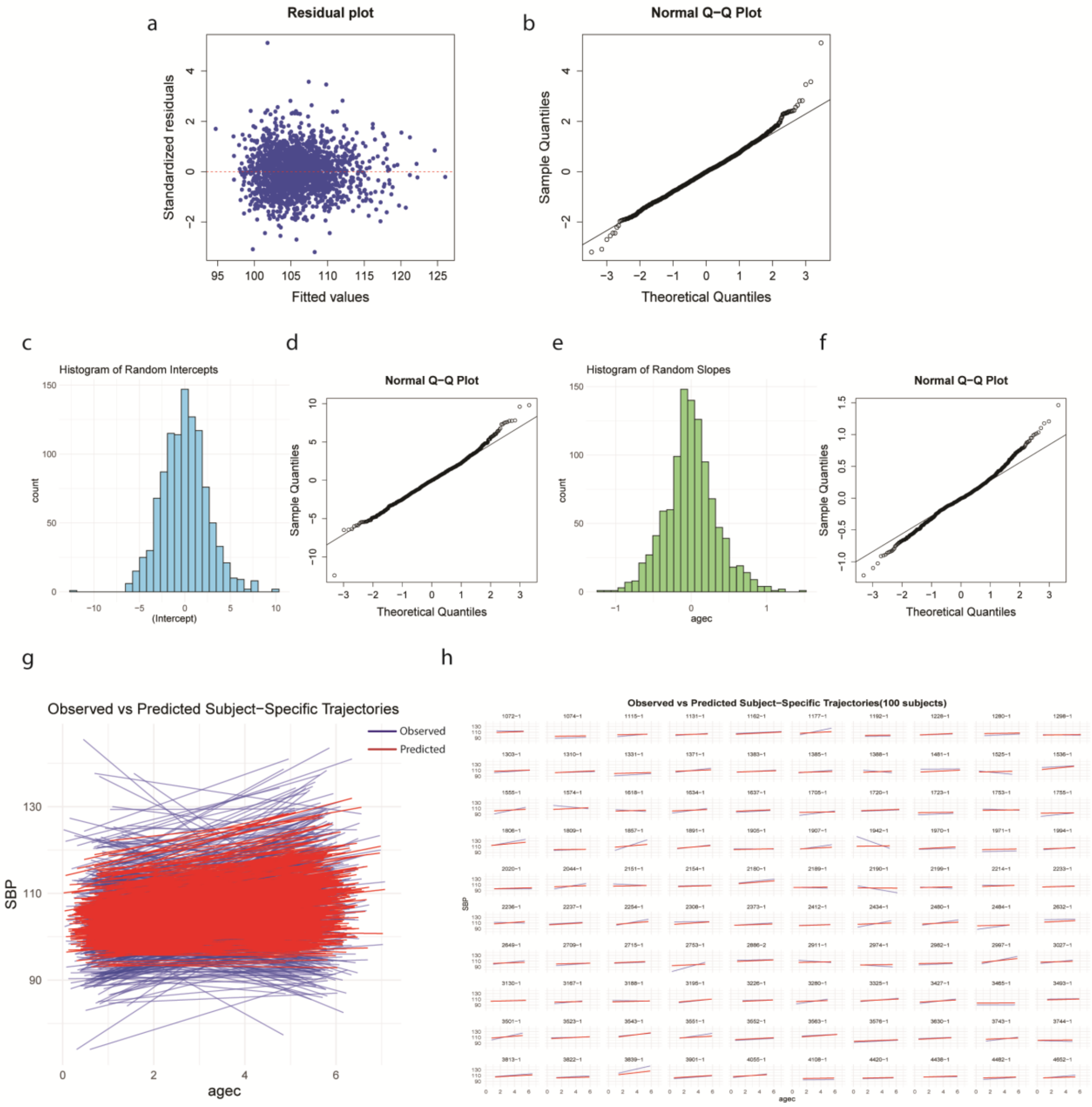

Figure S1. Diagnostic plots of the linear mixed model for SBP in prepuberty, with PRS included as a continuous variable. a. Residual plot (standardized conditional residuals vs. marginal fitted values); b. Normal Q-Q plot for standardized conditional residuals; c. Histogram of random intercepts; d. Normal Q-Q plot for random intercepts; e. Histogram of random slopes for centered age (agec); f. Normal Q-Q plot of random slopes for agec; g. Observed vs. Predicted SBP trajectories for all subjects; h. Observed vs. Predicted SBP trajectories for a randomly selected subset of 100 subjects.

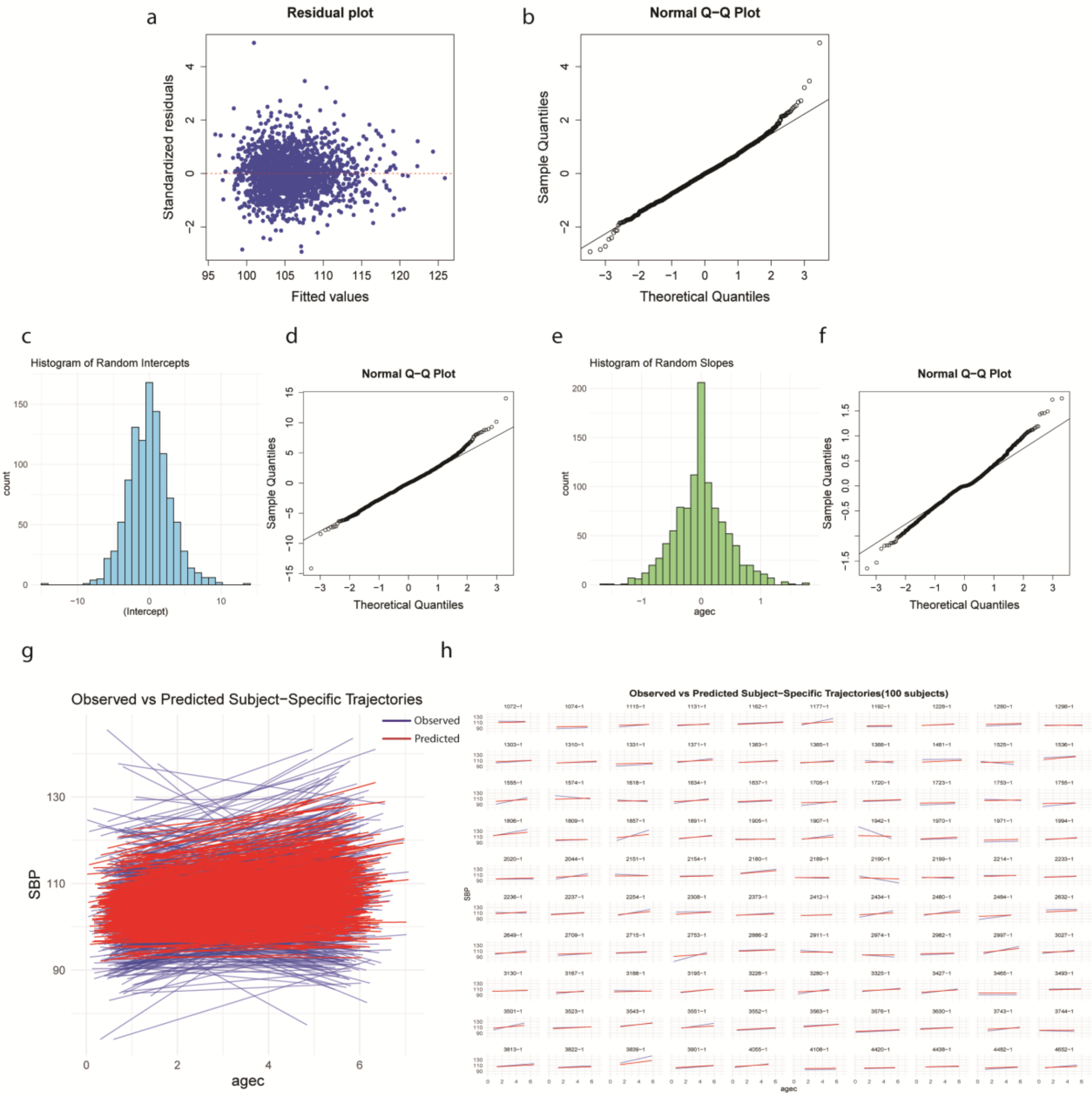

Figure S2. Diagnostic plots of the linear mixed model for SBP in prepuberty, with PRS included as a categorical variable. a. Residual plot (standardized conditional residuals vs. marginal fitted values); b. Normal Q-Q plot for standardized conditional residuals; c. Histogram of random intercepts; d. Normal Q-Q plot for random intercepts; e. Histogram of random slopes for centered age (agec); f. Normal Q-Q plot of random slopes for agec; g. Observed vs. Predicted SBP trajectories for all subjects; h. Observed vs. Predicted SBP trajectories for a randomly selected subset of 100 subjects.

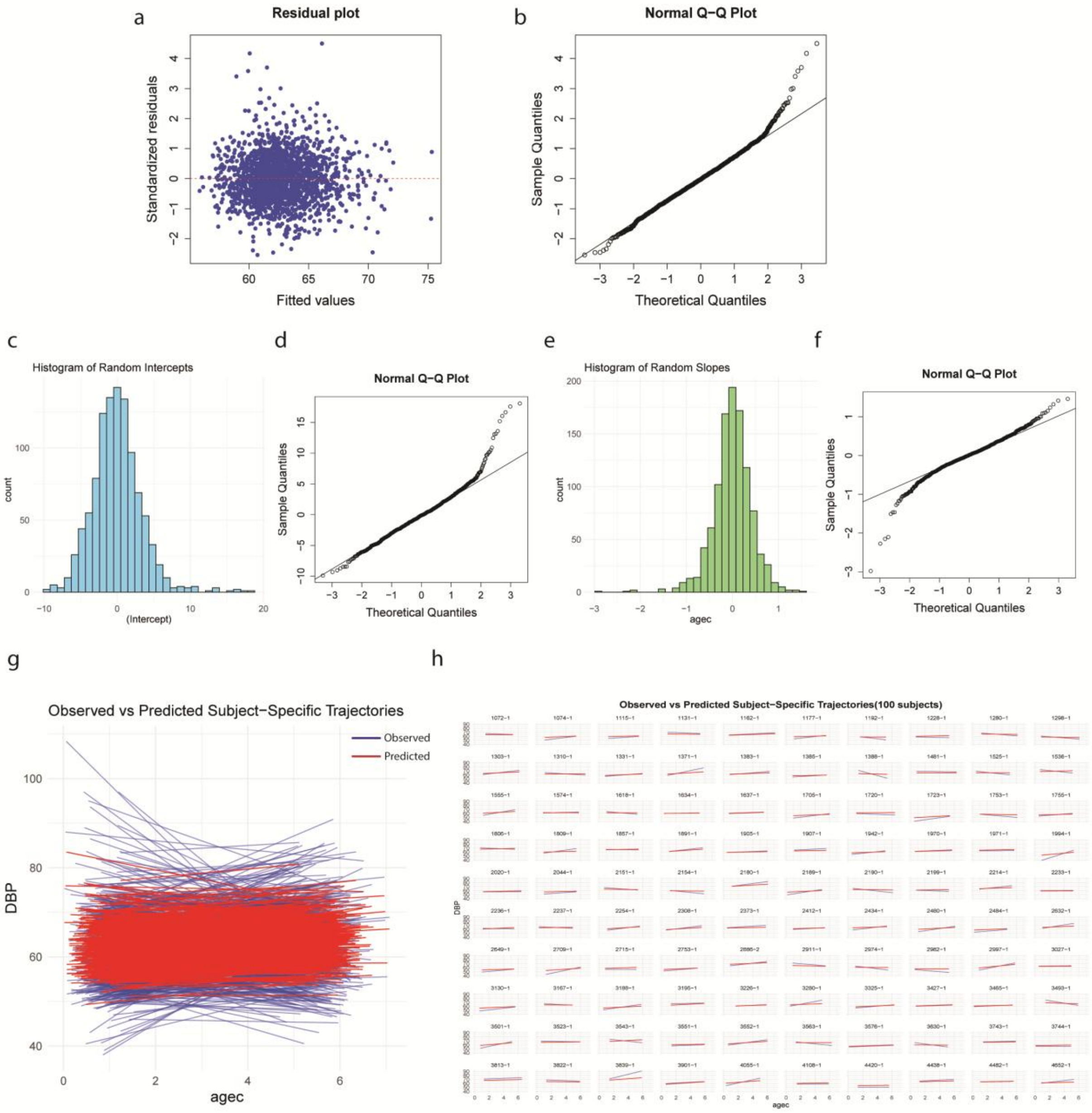

Figure S3. Diagnostic plots of the linear mixed model for DBP in prepuberty, with PRS included as a continuous variable. a. Residual plot (standardized conditional residuals vs. marginal fitted values); b. Normal Q-Q plot for standardized conditional residuals; c. Histogram of random intercepts; d. Normal Q-Q plot for random intercepts; e. Histogram of random slopes for centered age (agec); f. Normal Q-Q plot of random slopes for agec; g. Observed vs. Predicted DBP trajectories for all subjects; h. Observed vs. Predicted DBP trajectories for a randomly selected subset of 100 subjects.

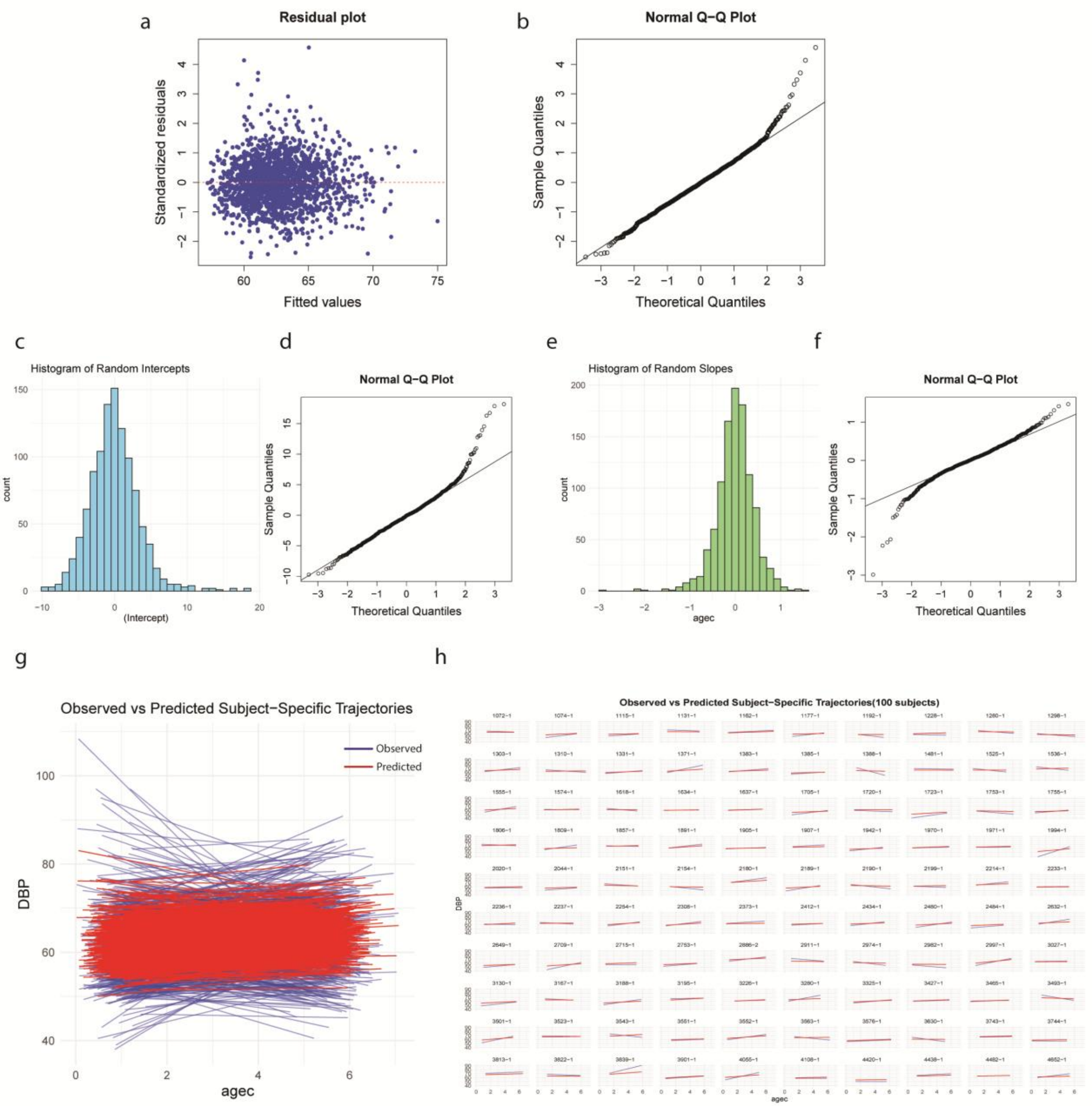

Figure S4. Diagnostic plots of the linear mixed model for DBP in prepuberty, with PRS included as a categorical variable. a. Residual plot (standardized conditional residuals vs. marginal fitted values); b. Normal Q-Q plot for standardized conditional residuals; c. Histogram of random intercepts; d. Normal Q-Q plot for random intercepts; e. Histogram of random slopes for centered age (agec); f. Normal Q-Q plot of random slopes for agec; g. Observed vs. Predicted DBP trajectories for all subjects; h. Observed vs. Predicted DBP trajectories for a randomly selected subset of 100 subjects.

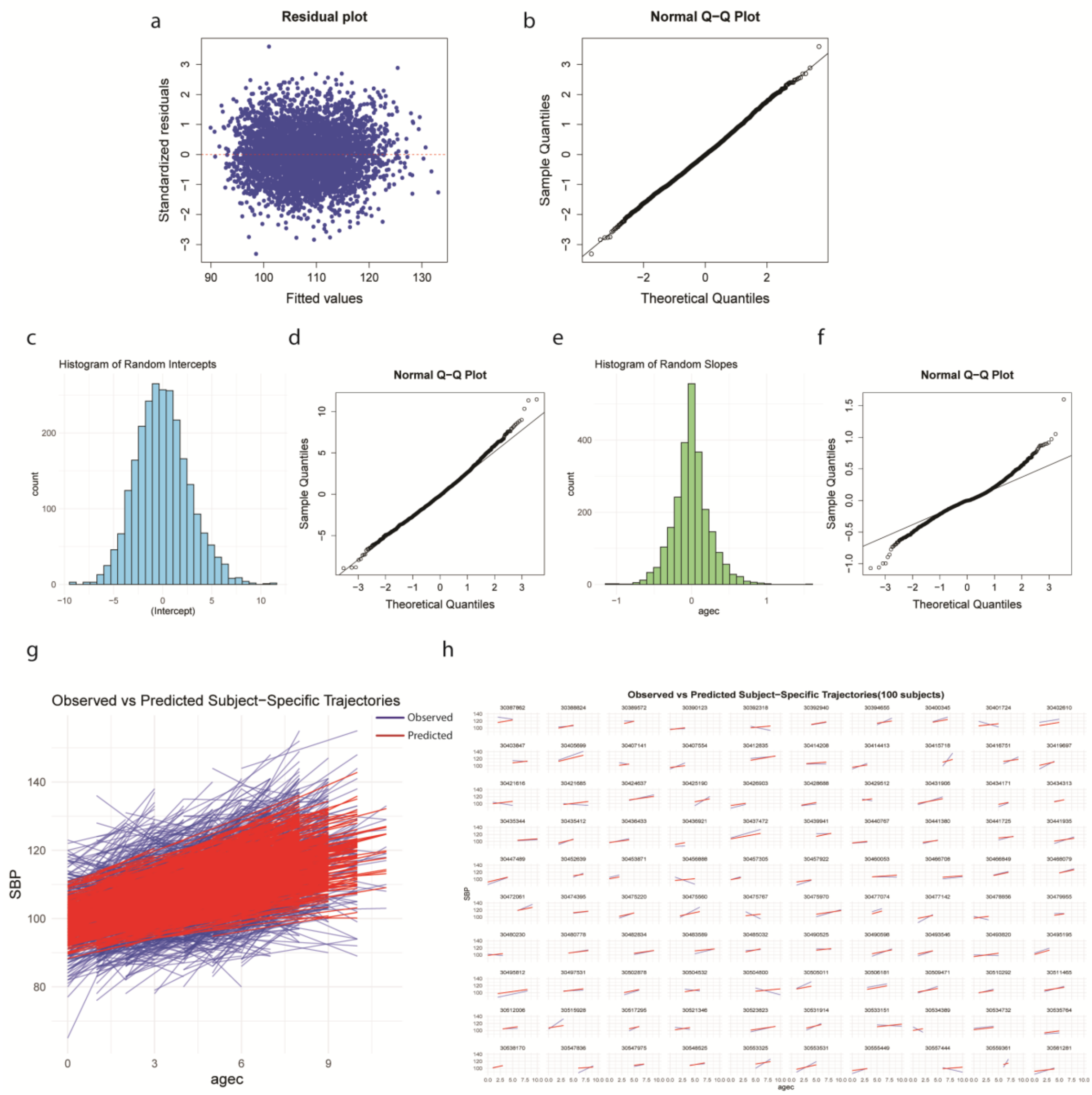

Figure S5. Diagnostic plots of the linear mixed model for SBP in puberty, with PRS included as a continuous variable. a. Residual plot (standardized conditional residuals vs. marginal fitted values); b. Normal Q-Q plot for standardized conditional residuals; c. Histogram of random intercepts; d. Normal Q-Q plot for random intercepts; e. Histogram of random slopes for centered age (agec); f. Normal Q-Q plot of random slopes for agec; g. Observed vs. Predicted SBP trajectories for all subjects; h. Observed vs. Predicted SBP trajectories for a randomly selected subset of 100 subjects.

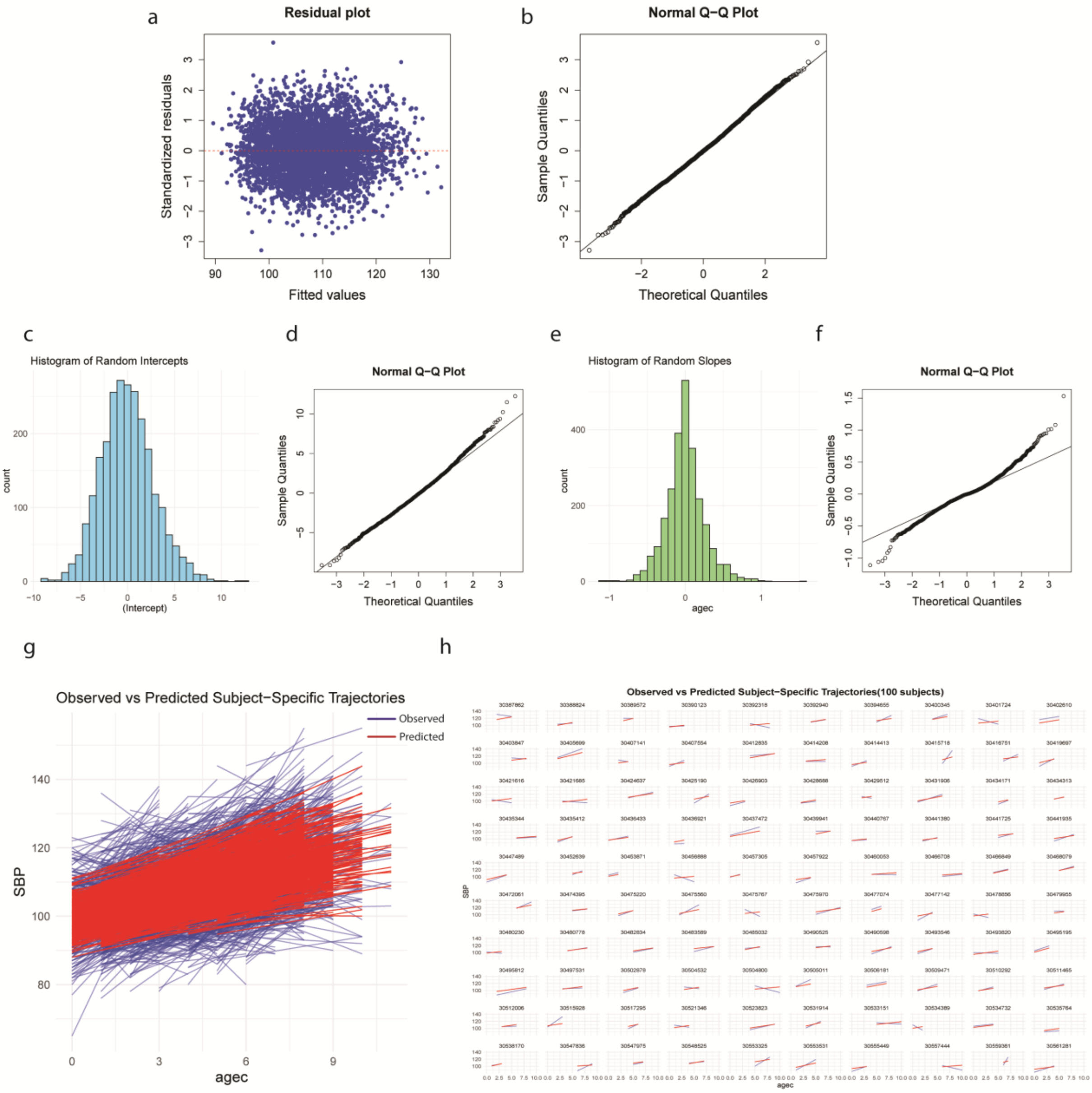

Figure S6. Diagnostic plots of the linear mixed model for SBP in puberty, with PRS included as a categorical variable. a. Residual plot (standardized conditional residuals vs. marginal fitted values); b. Normal Q-Q plot for standardized conditional residuals; c. Histogram of random intercepts; d. Normal Q-Q plot for random intercepts; e. Histogram of random slopes for centered age (agec); f. Normal Q-Q plot of random slopes for agec; g. Observed vs. Predicted SBP trajectories for all subjects; h. Observed vs. Predicted SBP trajectories for a randomly selected subset of 100 subjects.

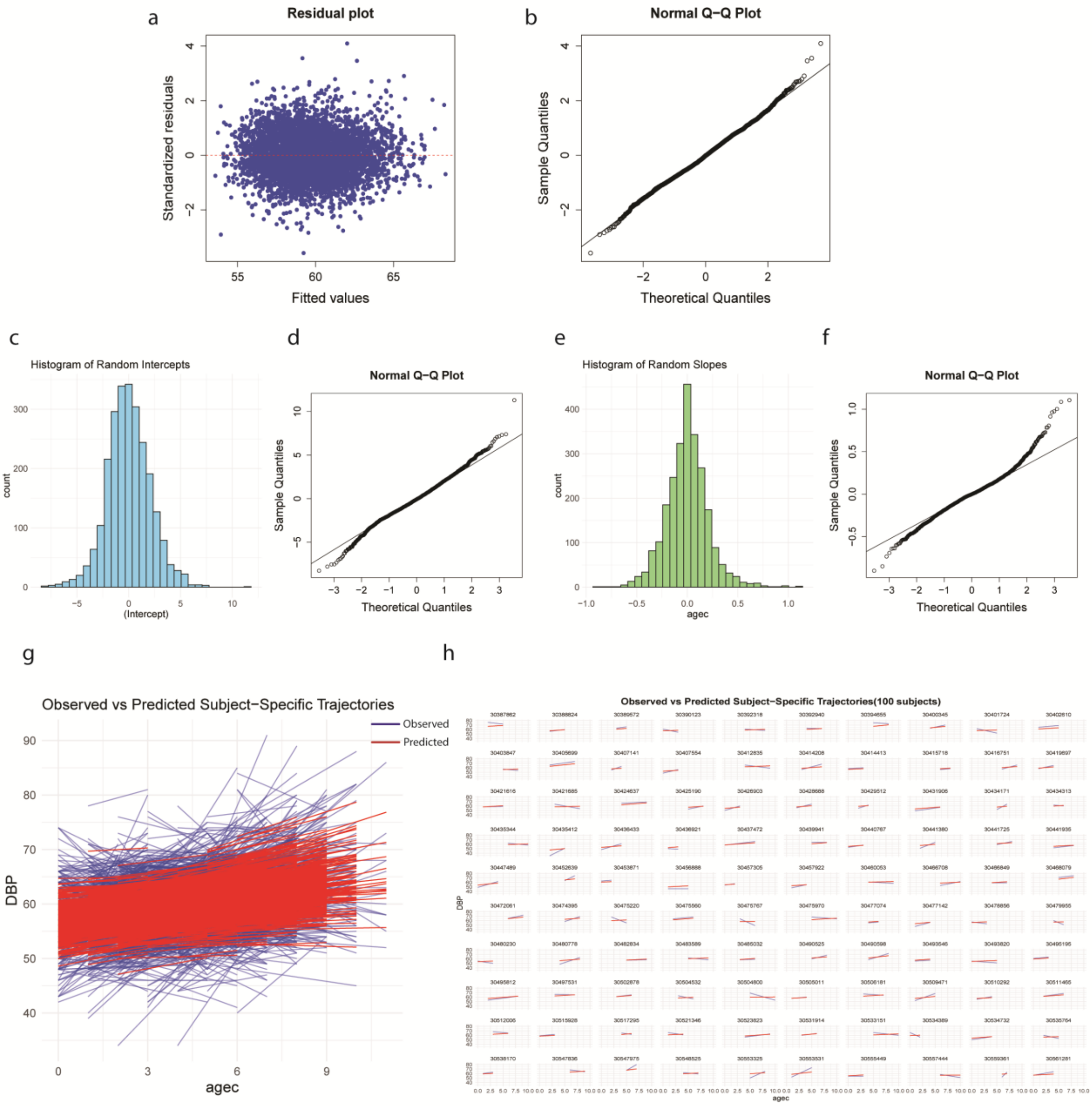

Figure S7. Diagnostic plots of the linear mixed model for DBP in puberty, with PRS included as a continuous variable. a. Residual plot (standardized conditional residuals vs. marginal fitted values); b. Normal Q-Q plot for standardized conditional residuals; c. Histogram of random intercepts; d. Normal Q-Q plot for random intercepts; e. Histogram of random slopes for centered age (agec); f. Normal Q-Q plot of random slopes for agec; g. Observed vs. Predicted DBP trajectories for all subjects; h. Observed vs. Predicted DBP trajectories for a randomly selected subset of 100 subjects.

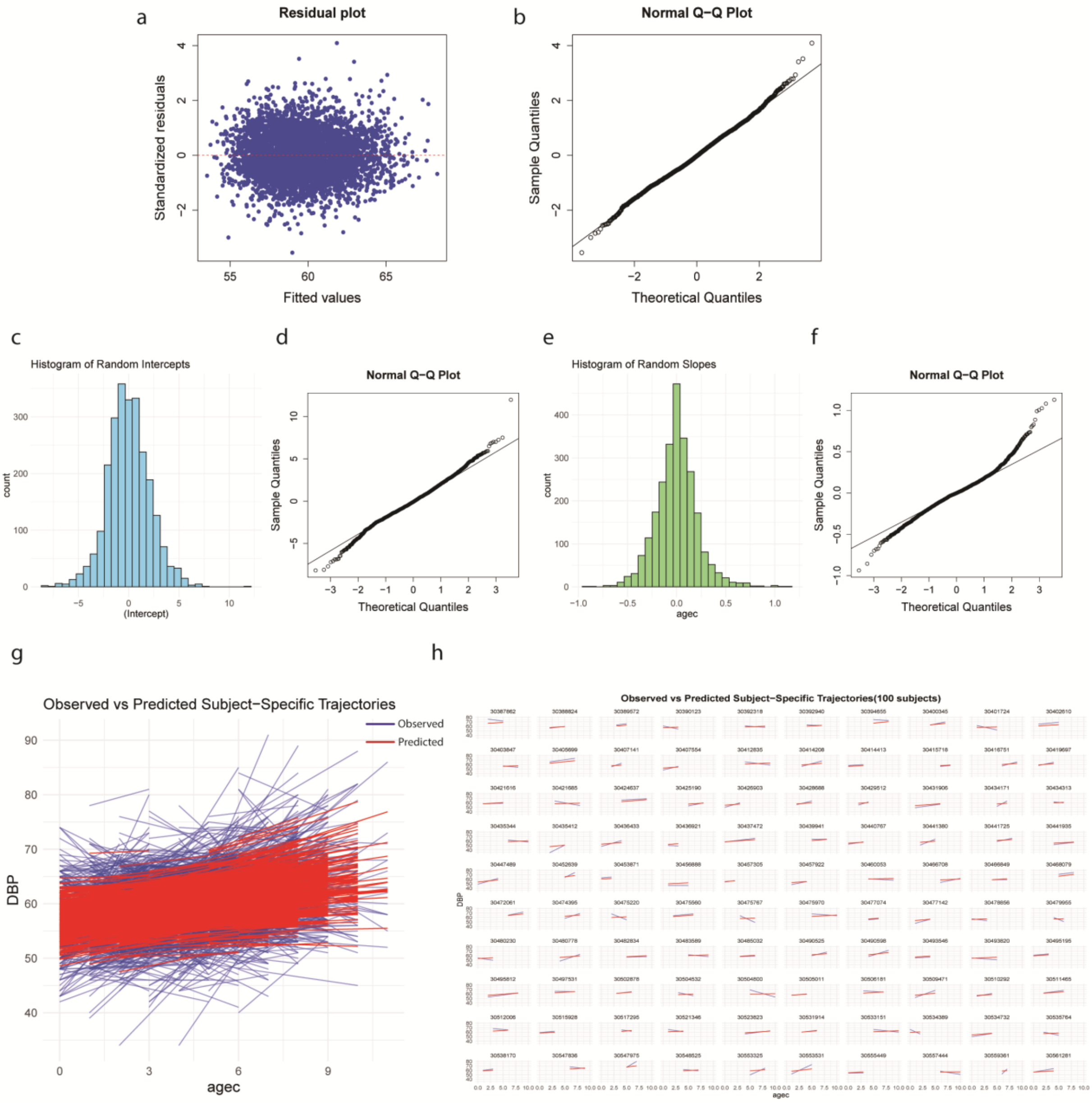

Figure S8. Diagnostic plots of the linear mixed model for DBP in puberty, with PRS included as a categorical variable. a. Residual plot (standardized conditional residuals vs. marginal fitted values); b. Normal Q-Q plot for standardized conditional residuals; c. Histogram of random intercepts; d. Normal Q-Q plot for random intercepts; e. Histogram of random slopes for centered age (agec); f. Normal Q-Q plot of random slopes for agec; g. Observed vs. Predicted DBP trajectories for all subjects; h. Observed vs. Predicted DBP trajectories for a randomly selected subset of 100 subjects.

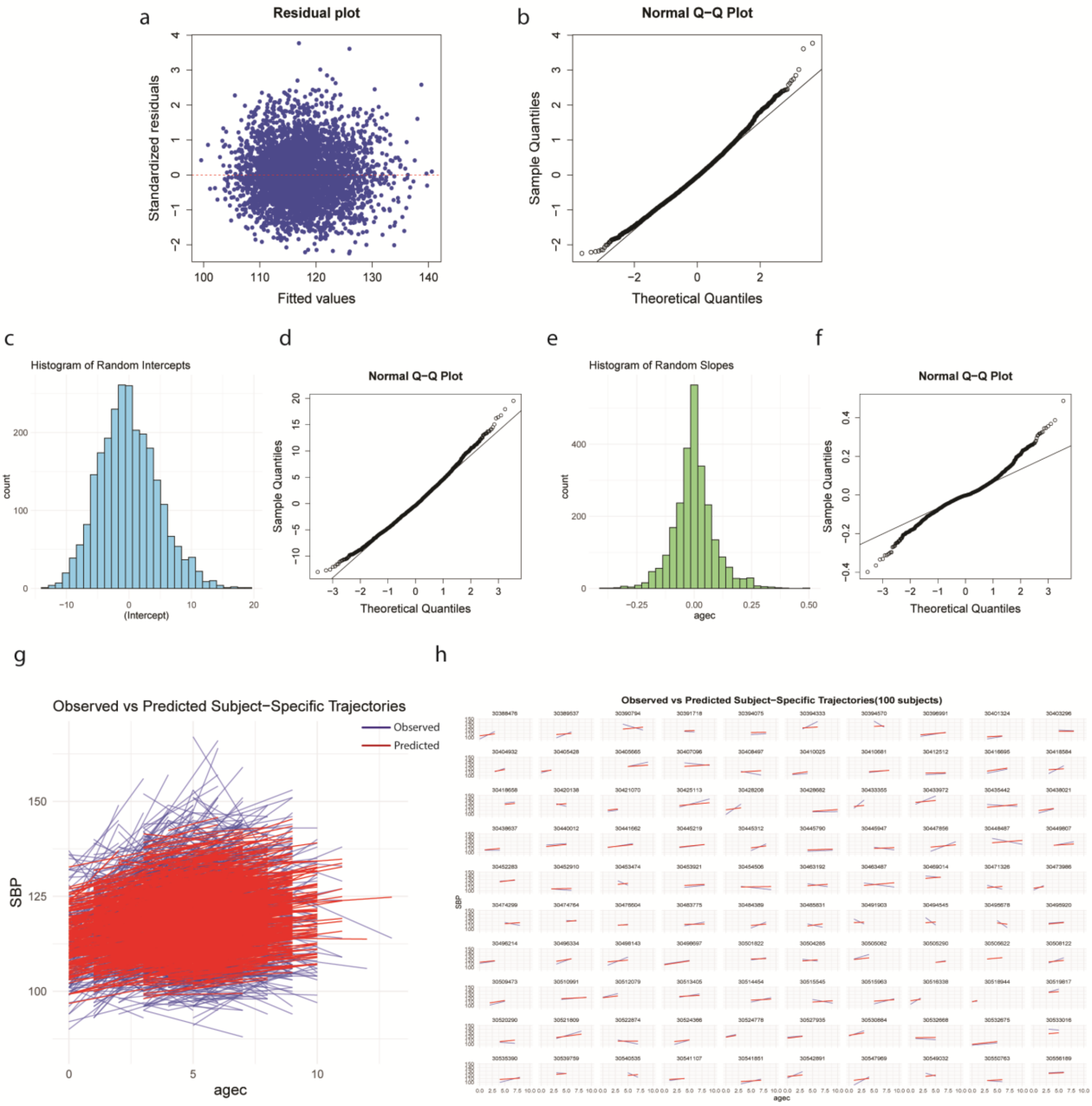

Figure S9. Diagnostic plots of the linear mixed model for SBP in postpuberty, with PRS included as a continuous variable. a. Residual plot (standardized conditional residuals vs. marginal fitted values); b. Normal Q-Q plot for standardized conditional residuals; c. Histogram of random intercepts; d. Normal Q-Q plot for random intercepts; e. Histogram of random slopes for centered age (agec); f. Normal Q-Q plot of random slopes for agec; g. Observed vs. Predicted SBP trajectories for all subjects; h. Observed vs. Predicted SBP trajectories for a randomly selected subset of 100 subjects.

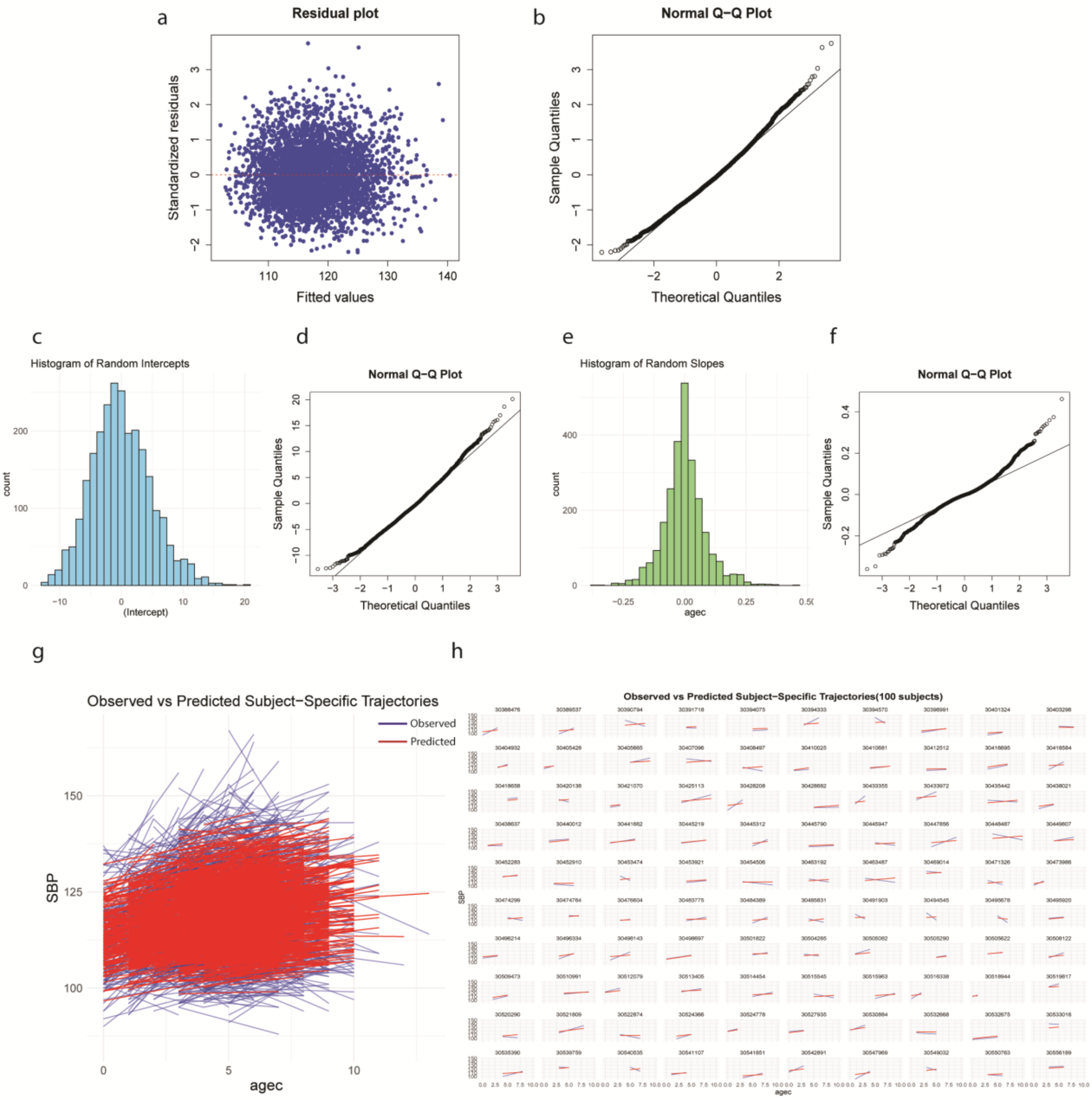

Figure S10. Diagnostic plots of the linear mixed model for SBP in postpuberty, with PRS included as a categorical variable. a. Residual plot (standardized conditional residuals vs. marginal fitted values); b. Normal Q-Q plot for standardized conditional residuals; c. Histogram of random intercepts; d. Normal Q-Q plot for random intercepts; e. Histogram of random slopes for centered age (agec); f. Normal Q-Q plot of random slopes for agec; g. Observed vs. Predicted SBP trajectories for all subjects; h. Observed vs. Predicted SBP trajectories for a randomly selected subset of 100 subjects.

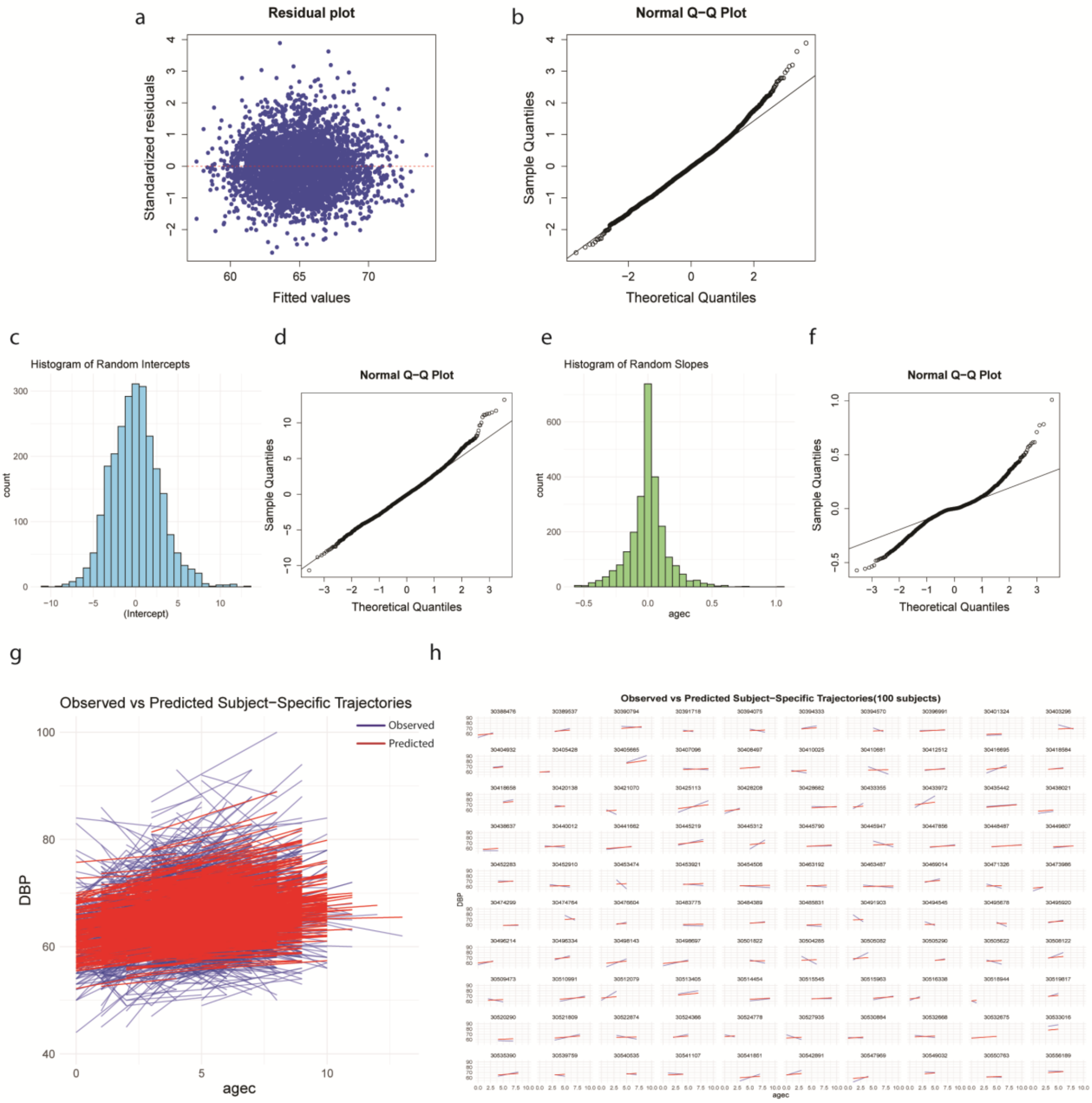

Figure S11. Diagnostic plots of the linear mixed model for DBP in postpuberty, with PRS included as a continuous variable. a. Residual plot (standardized conditional residuals vs. marginal fitted values); b. Normal Q-Q plot for standardized conditional residuals; c. Histogram of random intercepts; d. Normal Q-Q plot for random intercepts; e. Histogram of random slopes for centered age (agec); f. Normal Q-Q plot of random slopes for agec; g. Observed vs. Predicted DBP trajectories for all subjects; h. Observed vs. Predicted DBP trajectories for a randomly selected subset of 100 subjects.

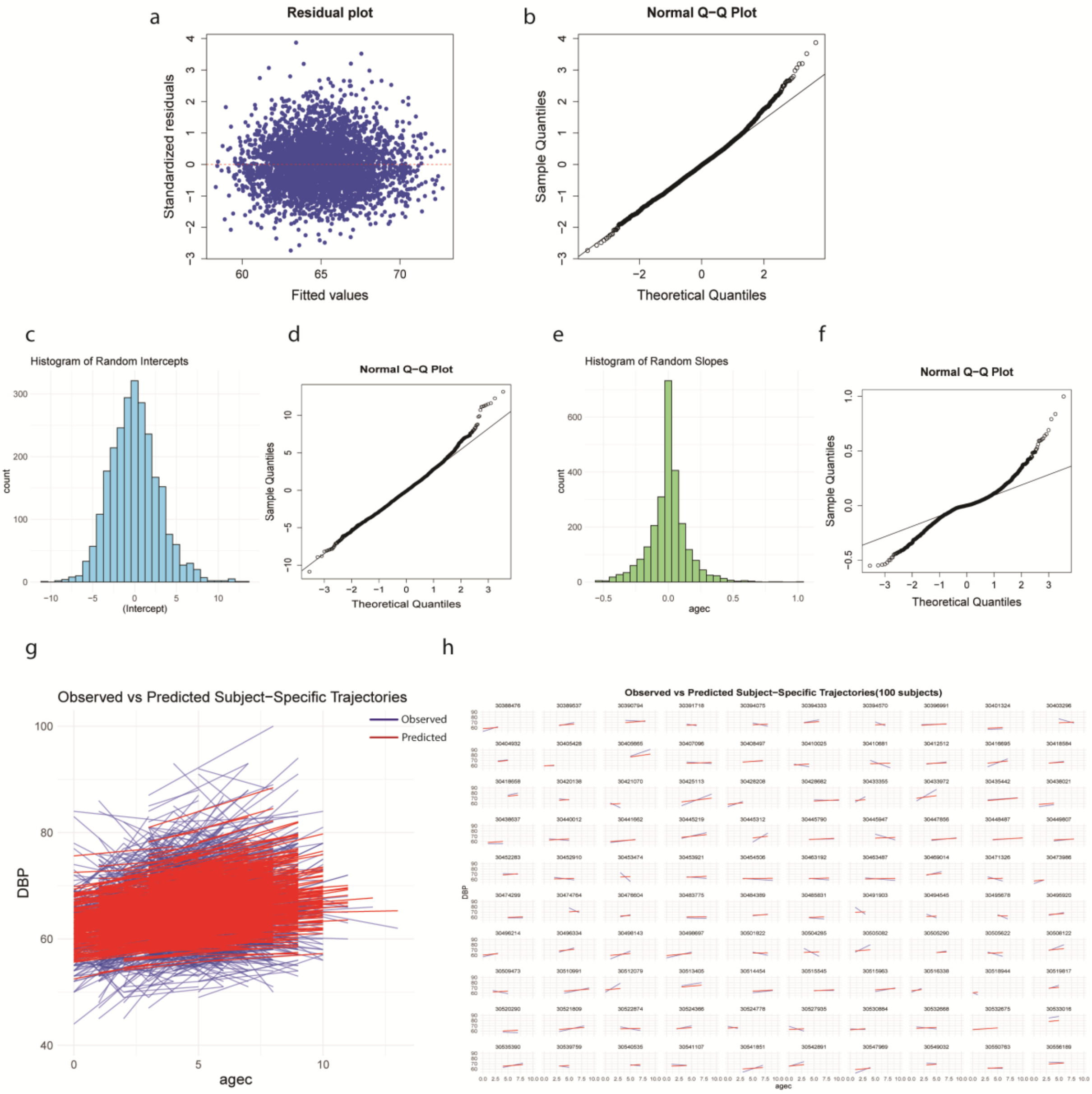

Figure S12. Diagnostic plots of the linear mixed model for DBP in postpuberty, with PRS included as a categorical variable. a. Residual plot (standardized conditional residuals vs. marginal fitted values); b. Normal Q-Q plot for standardized conditional residuals; c. Histogram of random intercepts; d. Normal Q-Q plot for random intercepts; e. Histogram of random slopes for centered age (agec); f. Normal Q-Q plot of random slopes for agec; g. Observed vs. Predicted DBP trajectories for all subjects; h. Observed vs. Predicted DBP trajectories for a randomly selected subset of 100 subjects.

Group authors

UMCG Genetics Lifelines Initiative (UGLI) group author: Lifelines Cohort Study

Raul Aguirre-Gamboa (1), Patrick Deelen (1), Lude Franke (1), Jan A Kuivenhoven (2), Esteban A Lopera Maya (1), Ilja M Nolte (3), Serena Sanna (1), Harold Snieder (3), Morris A Swertz (1), Peter M. Visscher (3,4), Judith M Vonk (3), Cisca Wijmenga (1)

1. *Department of Genetics, University of Groningen, University Medical Center Groningen, The Netherlands*
2. *Department of Pediatrics, University of Groningen, University Medical Center Groningen, The Netherlands*
3. *Department of Epidemiology, University of Groningen, University Medical Center Groningen, The Netherlands*
4. *Institute for Molecular Bioscience, The University of Queensland, Brisbane, Queensland, Australia.*

Authors & Contributors from the International Consortium of Blood Pressure (ICBP) associated with the 2018 Nature Genetics publication: "Genetic analysis of over one million people identifies 535 new loci associated with blood pressure traits" (Reference 7 in the main manuscript)

Evangelos Evangelou[1,2], Helen R Warren[3,4], He Gao[1,5], Georgios Ntritsos[2], Niki Dimou[2], Tonu Esko[16,17], Reedik Mägi[16], Lili Milani[16], Peter Almgren[18], Thibaud Boutin[19], Stéphanie Debette[20,21], Jun Ding[22], Franco Giulianini[23], Elizabeth G Holliday[24], Anne U Jackson[25], Ruifang Li-Gao[26], Wei-Yu Lin[27], Jian'an Luan[28], Massimo Mangino[29,30], Christopher Oldmeadow[24], Bram Peter Prins[31], Yong Qian[22], Muralidharan Sargurupremraj[21], Nabi Shah[32,33], Praveen Surendran[27], Sébastien Thériault[34,35], Niek Verweij[17,36,37], Sara M Willems[28], Jing-Hua Zhao[28], Philippe Amouyel[38], John Connell[39], Renée de Mutsert[26], Alex SF Doney[32], Martin Farrall[40,41], Cristina Menni[29], Andrew D Morris[42], Raymond Noordam[43], Guillaume Paré[34], Neil R Poulter[44], Denis C Shields[45], Alice Stanton[46], Simon Thom[47], Gonçalo Abecasis[48], Najaf Amin[49], Dan E Arking[50], Kristin L Ayers[51,52], Caterina M Barbieri[53], Chiara Batini[54], Joshua C Bis[55], Tineka Blake[54], Murielle Bochud[56], Michael Boehnke[25], Eric Boerwinkle[57], Dorret I Boomsma[58], Erwin P Bottinger[59], Peter S Braund[60,61], Marco Brumat[62], Archie Campbell[63,64], Harry Campbell[65], Aravinda Chakravarti[50], John C Chambers[1,5,66-68], Ganesh Chauhan[69], Marina Ciullo[70,71], Massimiliano Cocca[72], Francis Collins[73], Heather J Cordell[51], Gail Davies[74,75], Martin H de Borst[76], Eco J de Geus[58], Ian J Deary[74,75], Joris Deelen[77], Fabiola Del Greco M[78], Cumhur Yusuf Demirkale[79], Marcus Dörr[80,81], Georg B Ehret[50,82], Roberto Elosua[83,84], Stefan Enroth[85], A Mesut Erzurumluoglu[54], Teresa Ferreira[86,87], Mattias Frånberg[88-90], Oscar H Franco[91], Ilaria Gandin[62], Paolo Gasparini[62,72], Vilmantas Giedraitis[92], Christian Gieger[93-95], Giorgia Girotto[62,72], Anuj Goel[40,41], Alan J Gow[74,96], Vilmundur Gudnason[97,98], Xiuqing Guo[99], Ulf Gyllensten[85], Anders Hamsten[88,89], Tamara B Harris[100], Sarah E Harris[63,74], Catharina A Hartman[101], Aki S Havulinna[102,103], Andrew A Hicks[78], Edith Hofer[104,105], Albert Hofman[91,106], Jouke-Jan Hottenga[58], Jennifer E Huffman[19,107,108], Shih-Jen Hwang[107,108], Erik Ingelsson[109,110], Alan James[111,112], Rick Jansen[113], Marjo-Riitta Jarvelin[1,5,114-116], Roby Joehanes[107,117], Åsa Johansson[85], Andrew D Johnson[107,118], Peter K Joshi[65], Pekka Jousilahti[102], J Wouter Jukema[119], Antti Jula[102], Mika Kähönen[120,121], Sekar Kathiresan[17,36,122], Bernard D Keavney[123,124], Kay-Tee Khaw[125], Paul Knekt[102], Joanne Knight[126], Ivana Kolcic[127], Jaspal S Kooner[5,67,68,128], Seppo

Koskinen[102], Kati Kristiansson[102], Zoltan Kutalik[56,129], Maris Laan[130], Marty Larson[107], Lenore J Launer[100], Benjamin Lehne[1], Terho Lehtimäki[131,132], David CM Liewald[74,75], Li Lin[82], Lars Lind[133], Cecilia M Lindgren[40,87,134], YongMei Liu[135], Ruth JF Loos[28,59,136], Lorna M Lopez[74,137,138], Yingchang Lu[59], Leo-Pekka Lyytikäinen[131,132], Anubha Mahajan[40], Chrysovalanto Mamasoula[139], Jaume Marrugat[83], Jonathan Marten[19], Yuri Milaneschi[140], Anna Morgan[62], Andrew P Morris[40,141], Alanna C Morrison[142], Peter J Munson[79], Mike A Nalls[143,144], Priyanka Nandakumar[50], Christopher P Nelson[60,61], Teemu Niiranen[102,145], Ilja M Nolte[146], Teresa Nutile[70], Albertine J Oldehinkel[147], Ben A Oostra[49], Paul F O'Reilly[148], Elin Org[16], Sandosh Padmanabhan[64,149], Walter Palmas[150], Aarno Palotie[103,151,152], Alison Pattie[75], Brenda WJH Penninx[140], Markus Perola[102,103,153], Annette Peters[94,95,154], Ozren Polasek[127,155], Peter P Pramstaller[78,156,157], Quang Tri Nguyen[79], Olli T Raitakari[158,159], Rainer Rettig[161], Kenneth Rice[162], Paul M Ridker[23,163], Janina S Ried[94], Harriëtte Riese[147], Samuli Ripatti[103,164], Antonietta Robino[72], Lynda M Rose[23], Jerome I Rotter[99], Igor Rudan[165], Daniela Ruggiero[70,71], Yasaman Saba[166], Cinzia F Sala[53], Veikko Salomaa[102], Nilesh J Samani[60,61], Antti-Pekka Sarin[103], Reinhold Schmidt[104], Helena Schmidt[166], Nick Shrine[54], David Siscovick[167], Albert V Smith[97,98], Harold Snieder[146], Siim Sõber[130], Rossella Sorice[70], John M Starr[74,168], David J Stott[169], David P Strachan[170], Rona J Strawbridge[88,89], Johan Sundström[133], Morris A Swertz[171], Kent D Taylor[99], Alexander Teumer[81,172], Martin D Tobin[54], Maciej Tomaszewski[123,124], Daniela Toniolo[53], Michela Traglia[53], Stella Trompet[119,173], Jaakko Tuomilehto[174-177], Christophe Tzourio[21], André G Uitterlinden[91,178], Ahmad Vaez[146,179], Peter J van der Most[146], Cornelia M van Duijn[49], Germaine C Verwoert[91], Veronique Vitart[19], Uwe Völker[81,180], Peter Vollenweider[181], Dragana Vuckovic[62,182], Hugh Watkins[40,41], Sarah H Wild[183], Gonneke Willemsen[58], James F Wilson[19,65], Alan F Wright[19], Jie Yao[99], Tatijana Zemunik[184], Weihua Zhang[1,67], John R Attia[24], Adam S Butterworth[27,185], Daniel I Chasman[23,163], David Conen[186,187], Francesco Cucca[188,189], John Danesh[27,185], Caroline Hayward[19], Joanna MM Howson[27], Markku Laakso[190], Edward G Lakatta[191], Claudia Langenberg[28], Olle Melander[18], Dennis O Mook-Kanamori[26,192], Colin NA Palmer[32], Lorenz Risch[193-195], Robert A Scott[28], Rodney J Scott[24], Peter Sever[128], Tim D Spector[29], Pim van der Harst[196], Nicholas J Wareham[28], Eleftheria Zeggini[31], Daniel Levy[107,118], Patricia B Munroe[3,4], Christopher Newton-Cheh[134,197,198], Morris J Brown[3,4], Andres Metspalu[16], Bruce M. Psaty[201,202], Louise V Wain[54], Paul Elliott[1,5,203-205], Mark J Caulfield[3,4]

1. Department of Epidemiology and Biostatistics, Imperial College London, London, UK.
2. Department of Hygiene and Epidemiology, University of Ioannina Medical School, Ioannina, Greece.
3. William Harvey Research Institute, Barts and The London School of Medicine and Dentistry, Queen Mary University of London, London, UK.
4. National Institute for Health Research, Barts Cardiovascular Biomedical Research Center, Queen Mary University of London, London, UK.
5. MRC-PHE Centre for Environment and Health, Imperial College London, London, UK.

7. Division of Epidemiology, Department of Medicine, Institute for Medicine and Public Health, Vanderbilt Genetics Institute, Vanderbilt University Medical Center, Tennessee Valley Healthcare System (626)/Vanderbilt University, Nashville, TN, USA.
8. Vanderbilt Genetics Institute, Vanderbilt Epidemiology Center, Department of Obstetrics and Gynecology, Vanderbilt University Medical Center; Tennessee Valley Health Systems VA, Nashville, TN, USA.
9. Department of Epidemiology, Emory University Rollins School of Public Health, Atlanta, GA, USA.

10. Department of Biomedical Informatics, Emory University School of Medicine, Atlanta, GA, USA.
11. Massachusetts Veterans Epidemiology Research and Information Center (MAVERIC), VA Boston Healthcare System, Boston, USA.
12. Division of Aging, Department of Medicine, Brigham and Women's Hospital, Boston, MA, Department of Medicine, Harvard Medical School, Boston, MA, USA.
13. Atlanta VAMC and Emory Clinical Cardiovascular Research Institute, Atlanta, GA, USA.
14. VA Palo Alto Health Care System; Division of Cardiovascular Medicine, Stanford University School of Medicine, CA, USA.
15. Nephrology Section, Memphis VA Medical Center and University of Tennessee Health Science Center, Memphis, TN, USA.
16. Estonian Genome Center, University of Tartu, Tartu, Estonia.
17. Program in Medical and Population Genetics, Broad Institute of Harvard and MIT, Cambridge, MA, USA.
18. Department Clinical Sciences, Malmö, Lund University, Malmö, Sweden.
19. MRC Human Genetics Unit, MRC Institute of Genetics and Molecular Medicine, University of Edinburgh, Western General Hospital, Edinburgh, Scotland, UK
20. Department of Neurology, Bordeaux University Hospital, Bordeaux, France.
21. Univ. Bordeaux, Inserm, Bordeaux Population Health Research Center, CHU Bordeaux, Bordeaux, France.
22. Laboratory of Genetics and Genomics, NIA/NIH , Baltimore, MD, USA.
23. Division of Preventive Medicine, Brigham and Women's Hospital, Boston, MA, USA.
24. Hunter Medical Reseach Institute and Faculty of Health, University of Newcastle, New Lambton Heights, New South Wales, Australia.
25. Department of Biostatistics and Center for Statistical Genetics, University of Michigan, Ann Arbor, MI, USA.
26. Department of Clinical Epidemiology, Leiden University Medical Center, Leiden, the Netherlands.
27. MRC/BHF Cardiovascular Epidemiology Unit, Department of Public Health and Primary Care, University of Cambridge, Cambridge, UK.
28. MRC Epidemiology Unit, University of Cambridge School of Clinical Medicine, Cambridge, UK.
29. Department of Twin Research and Genetic Epidemiology, Kings College London, London, UK.
30. NIHR Biomedical Research Centre at Guy's and St Thomas' Foundation Trust, London, UK.
31. Wellcome Trust Sanger Institute, Hinxton, UK.
32. Division of Molecular and Clinical Medicine, School of Medicine, University of Dundee, UK.
33. Department of Pharmacy, COMSATS Institute of Information Technology, Abbottabad, Pakistan.
34. Department of Pathology and Molecular Medicine, McMaster University, Hamilton, Canada.
35. Institut universitaire de cardiologie et de pneumologie de Québec-Université Laval, , Quebec City, Canada.
36. Cardiovascular Research Center and Center for Human Genetic Research, Massachusetts General Hospital, Boston, Massachusetts, MA, USA.

37. University of Groningen, University Medical Center Groningen, Department of Cardiology, Groningen, The Netherlands.
38. University of Lille, Inserm, Centre Hosp. Univ Lille, Institut Pasteur de Lille, UMR1167 - RID-AGE - Risk factors and molecular determinants of aging-related diseases, Epidemiology and Public Health Department, Lille, France.
39. University of Dundee, Ninewells Hospital & Medical School, Dundee, , UK.
40. Wellcome Trust Centre for Human Genetics, University of Oxford, Oxford, UK.
41. Division of Cardiovascular Medicine, Radcliffe Department of Medicine, University of Oxford, Oxford, UK.
42. Usher Institute of Population Health Sciences and Informatics, University of Edinburgh, UK.
43. Department of Internal Medicine, Section Gerontology and Geriatrics, Leiden University Medical Center, Leiden, The Netherlands.
44. Imperial Clinical Trials Unit, Stadium House, 68 Wood Lane, London, UK.
45. School of Medicine, University College Dublin, Ireland.
46. Molecular and Cellular Therapeutics, Royal College of Surgeons in Ireland, Dublin, Ireland.
47. International Centre for Circulatory Health, Imperial College London, London, UK.
48. Center for Statistical Genetics, Dept. of Biostatistics, SPH II, Washington Heights, Ann Arbor, MI, USA.
49. Genetic Epidemiology Unit, Department of Epidemiology, Erasmus MC, Rotterdam, the Netherlands.
50. Center for Complex Disease Genomics, McKusick-Nathans Institute of Genetic Medicine, Johns Hopkins University School of Medicine, Baltimore, MD, USA.
51. Institute of Genetic Medicine, Newcastle University, Newcastle upon Tyne, UK.
52. Sema4, a Mount Sinai venture, Stamford, CT, USA.
53. Division of Genetics and Cell Biology, San Raffaele Scientific Institute, Milano, Italy.
54. Department of Health Sciences, University of Leicester, Leicester, UK.
55. Cardiovascular Health Research Unit, Department of Medicine, University of Washington, Seattle, WA, USA.
56. Institute of Social and Preventive Medicine, University Hospital of Lausanne, Lausanne, Switzerland.
57. Human Genetics Center, School of Public Health, The University of Texas Health Science Center at Houston and Human Genome Sequencing Center, Baylor College of Medicine, One Baylor Plaza, Houston, TX, USA.
58. Department of Biological Psychology, Vrije Universiteit Amsterdam, EMGO+ institute, VU University medical center, Amsterdam, the Netherlands.
59. The Charles Bronfman Institute for Personalized Medicine, Icachn School of Medicine at Mount Sinai, NY, USA.
60. Department of Cardiovascular Sciences, University of Leicester, Leicester, UK.
61. NIHR Leicester Biomedical Research Centre, Glenfield Hospital, Groby Road, Leicester, UK.
62. Department of Medical, Surgical and Health Sciences, University of Trieste, , Trieste, Italy.
63. Medical Genetics Section, Centre for Genomic and Experimental Medicine, Institute of Genetics and Molecular Medicine, University of Edinburgh, Edinburgh, UK.

64. Generation Scotland, Centre for Genomic and Experimental Medicine, University of Edinburgh, Edinburgh, UK.
65. Centre for Global Health Research, Usher Institute of Population Health Sciences and Informatics, University of Edinburgh, Edinburgh, Scotland, UK
66. Lee Kong Chian School of Medicine, Nanyang Technological University, Singapore, Singapore.
67. Department of Cardiology, Ealing Hospital, Middlesex, UK.
68. Imperial College Healthcare NHS Trust, London, UK.
69. Centre for Brain Research, Indian Institute of Science, Bangalore, India.
70. Institute of Genetics and Biophysics "A. Buzzati-Traverso", CNR, Napoli, Italy.
71. IRCCS Neuromed, Pozzilli, Isernia, Italy.
72. Institute for Maternal and Child Health IRCCS Burlo Garofolo, Trieste, Italy.
73. Medical Genomics and Metabolic Genetics Branch, National Human Genome Research Institute, NIH, Bethesda, MD, USA.
74. Centre for Cognitive Ageing and Cognitive Epidemiology, University of Edinburgh, 7 George Square, Edinburgh, UK.
75. Department of Psychology, University of Edinburgh, 7 George Square, Edinburgh, UK.
76. Department of Internal Medicine, Division of Nephrology, University of Groningen, University Medical Center Groningen, Groningen, The Netherlands.
77. Department of Molecular Epidemiology, Leiden University Medical Center, Leiden, the Netherlands.
78. Institute for Biomedicine, Eurac Research, Bolzano, Italy - Affiliated Institute of the University of Lübeck, Lübeck, Germany.
79. Mathematical and Statistical Computing Laboratory, Office of Intramural Research, Center for Information Technology, National Institutes of Health, Bethesda, MD, USA.
80. Department of Internal Medicine B, University Medicine Greifswald, Greifswald, Germany.
81. DZHK (German Centre for Cardiovascular Research), partner site Greifswald, Greifswald, Germany.
82. Cardiology, Department of Medicine, Geneva University Hospital, Geneva, Switzerland.
83. CIBERCV & Cardiovascular Epidemiology and Genetics, IMIM. Dr Aiguader 88, Barcelona, Spain.
84. Faculty of Medicine, Universitat de Vic-Central de Catalunya, Vic, Spain.
85. Department of Immunology, Genetics and Pathology, Uppsala Universitet, Science for Life Laboratory, Uppsala, Sweden.
86. Wellcome Centre for Human Genetics, University of Oxford, Roosevelt Drive, Oxford, UK.
87. Big Data Institute, Li Ka Shing Center for Health for Health Information and Discovery, Oxford University, Old Road, Oxford, UK.
88. Cardiovascular Medicine Unit, Department of Medicine Solna, Karolinska Institutet, Stockholm, Sweden.
89. Centre for Molecular Medicine, L8:03, Karolinska Universitetsjukhuset, Solna, Sweden.
90. Department of Numerical Analysis and Computer Science, Stockholm University, Stockholm, Sweden.
91. Department of Epidemiology, Erasmus MC, Rotterdam, the Netherlands.

92. Department of Public Health and Caring Sciences, Geriatrics, Uppsala, Sweden.
93. Research Unit of Molecular Epidemiology, Helmholtz Zentrum München, German Research Center for Environmental Health, Neuherberg, Germany.
94. Institute of Epidemiology, Helmholtz Zentrum München, German Research Center for Environmental Health, Neuherberg, Germany.
95. German Center for Diabetes Research (DZD e.V.), Neuherberg, Germany.
96. Department of Psychology, School of Social Sciences, Heriot-Watt University, Edinburgh, UK.
97. Faculty of Medicine, University of Iceland, Reykjavik, Iceland.
98. Icelandic Heart Association, Kopavogur, Iceland.
99. The Institute for Translational Genomics and Population Sciences, Department of Pediatrics, LABioMed at Harbor-UCLA Medical Center, Torrance, CA, USA.
100. Intramural Research Program, Laboratory of Epidemiology, Demography, and Biometry, National Institute on Aging, Bethesda, MD, USA.
101. Department of Psychiatry, University of Groningen, University Medical Center Groningen, Groningen, The Netherlands.
102. Department of Public Health Solutions, National Institute for Health and Welfare (THL), Helsinki, Finland.
103. Institute for Molecular Medicine Finland (FIMM), University of Helsinki, Helsinki, Finland.
104. Clinical Division of Neurogeriatrics, Department of Neurology, Medical University of Graz, Graz, Austria.
105. Institute for Medical Informatics, Statistics and Documentation, Medical University of Graz, Graz, Austria.
106. Department of Epidemiology, Harvard T.H. Chan School of Public Health, Boston, MA, USA.
107. National Heart, Lung and Blood Institute's Framingham Heart Study, Framingham, MA, USA.
108. The Population Science Branch, Division of Intramural Research, National Heart Lung and Blood Institute national Institute of Health, Bethesda, MD, USA.
109. Department of Medical Sciences, Molecular Epidemiology and Science for Life Laboratory, Uppsala University, Uppsala, Sweden.
110. Division of Cardiovascular Medicine, Department of Medicine, Stanford University School of Medicine, Stanford, CA USA.
111. Department of Pulmonary Physiology and Sleep, Sir Charles Gairdner Hospital, Hospital Avenue, Nedlands, Australia.
112. School of Medicine and Pharmacology, University of Western Australia.
113. Department of Psychiatry, VU University Medical Center, Amsterdam Neuroscience, Amsterdam, the Netherlands.
114. Biocenter Oulu, University of Oulu, Oulu, Finland.
115. Center For Life-course Health Research, University of Oulu, Oulu Finland.
116. Unit of Primary Care, Oulu University Hospital, Oulu, Oulu, Finland.
117. Hebrew SeniorLife, Harvard Medical School, Boston, MA, USA.
118. Population Sciences Branch, National Heart, Lung and Blood Institute, National Institutes of Health, Bethesda, MD, USA.
119. Department of Cardiology, Leiden University Medical Center, Leiden, the Netherlands.
120. Department of Clinical Physiology, Tampere University Hospital, Tampere, Finland.

121. Department of Clinical Physiology, Finnish Cardiovascular Research Center - Tampere, Faculty of Medicine and Life Sciences, University of Tampere, Tampere, Finland.
122. Broad Institute of the Massachusetts Institute of Technology and Harvard University, Cambridge, MA, USA.
123. Division of Cardiovascular Sciences, Faculty of Biology, Medicine and Health, The University of Manchester, Manchester, UK.
124. Division of Medicine, Manchester University NHS Foundation Trust, Manchester Academic Health Science Centre, Manchester, UK
125. Department of Public Health and Primary Care, Institute of Public Health, University of Cambridge, Cambridge, UK.
126. Data Science Institute and Lancaster Medical School, Lancaster, UK.
127. Department of Public Health, Faculty of Medicine, University of Split, Croatia.
128. National Heart and Lung Institute, Imperial College London, London, UK.
129. Swiss Institute of Bioinformatics, Lausanne, Switzerland.
130. Institute of Biomedicine and Translational Medicine, University of Tartu, Tartu, Estonia.
131. Department of Clinical Chemistry, Fimlab Laboratories, Tampere, Finland.
132. Department of Clinical Chemistry, Finnish Cardiovascular Research Center - Tampere, Faculty of Medicine and Life Sciences, University of Tampere, Tampere, Finland
133. Department of Medical Sciences, Cardiovascular Epidemiology, Uppsala University, Uppsala, Sweden.
134. Program in Medical and Population Genetics, Broad Institute, Cambridge, MA, USA.
135. Division of Public Health Sciences, Wake Forest School of Medicine, Winston-Salem, NC, USA.
136. Mindich Child health Development Institute, The Icahn School of Medicine at Mount Sinai, New York, NY, USA.
137. Department of Psychiatry, Royal College of Surgeons in Ireland, Education and Research Centre, Beaumont Hospital, Dublin, Ireland.
138. University College Dublin, UCD Conway Institute, Centre for Proteome Research, UCD, Belfield, Dublin, Ireland.
139. Institute of Health and Society, Newcastle University, Newcastle upon Tyne, UK.
140. Department of Psychiatry, Amsterdam Public Health and Amsterdam Neuroscience, VU University Medical Center/GGZ inGeest, Amsterdam, The Netherlands.
141. Department of Biostatistics, University of Liverpool, Block F, Waterhouse Building, Liverpool, UK.
142. Department of Epidemiology, Human Genetics and Environmental Sciences, School of Public Health, University of Texas Health Science Center at Houston, Houston, TX, USA.
143. Data Tecnica International, Glen Echo, MD, USA.
144. Laboratory of Neurogenetics, National Institute on Aging, Bethesda, USA.
145. Department of Medicine, Turku University Hospital and University of Turku, Finland.
146. Department of Epidemiology, University of Groningen, University Medical Center Groningen, Groningen, The Netherlands.
147. Interdisciplinary Center Psychopathology and Emotion regulation (ICPE), University of Groningen, University Medical Center Groningen, Groningen, The Netherlands.
148. SGDP Centre, Institute of Psychiatry, Psychology and Neuroscience, King's College London, London, UK.

149. British Heart Foundation Glasgow Cardiovascular Research Centre, Institute of Cardiovascular and Medical Sciences, College of Medical, Veterinary and Life Sciences, University of Glasgow, Glasgow, UK.
150. Department of Medicine, Columbia University Medical Center, New York, NY, USA.
151. Analytic and Translational Genetics Unit, Department of Medicine, Department of Neurology and Department of Psychiatry Massachusetts General Hospital, Boston, MA, USA.
152. The Stanley Center for Psychiatric Research and Program in Medical and Population Genetics, The Broad Institute of MIT and Harvard, Cambridge, MA, USA.
153. University of Tartu, Tartu, Estonia.
154. German Center for Cardiovascular Disease Research (DZHK), partner site Munich, Neuherberg, Germany.
155. Psychiatric hospital “Sveti Ivan”, Zagreb, Croatia.
156. Department of Neurology, General Central Hospital, Bolzano, Italy.
157. Department of Neurology, University of Lübeck, Lübeck, Germany.
158. Department of Clinical Physiology and Nuclear Medicine, Turku University Hospital, Turku, Finland.
159. Research Centre of Applied and Preventive Cardiovascular Medicine, University of Turku, Turku, Finland.
161. Institute of Physiology, University Medicine Greifswald, Karlsburg, Germany.
162. Department of Biostatistics University of Washington, Seattle, WA, USA.
163. Harvard Medical School, Boston MA.
164. Public health, Faculty of Medicine, University of Helsinki, Finland
165. Centre for Global Health Research, Usher Institute of Population Health Sciences and Informatics, University of Edinburgh, Scotland, UK.
166. Gottfried Schatz Research Center for Cell Signaling, Metabolism & Aging, Molecular Biology and Biochemistry, Medical University of Graz, Graz, Austria.
167. The New York Academy of Medicine, New York, NY, USA.
168. Alzheimer Scotland Dementia Research Centre, University of Edinburgh, Edinburgh, UK.
169. Institute of Cardiovascular and Medical Sciences, Faculty of Medicine, University of Glasgow, United Kingdom.
170. Population Health Research Institute, St George's, University of London, London, UK.
171. Department of Genetics, University of Groningen, University Medical Center Groningen, Groningen, The Netherlands.
172. Institute for Community Medicine, University Medicine Greifswald, Greifswald, Germany.
173. Department of Gerontology and Geriatrics, Leiden University Medical Center, Leiden, the Netherlands.
174. Dasman Diabetes Institute, Dasman, Kuwait.
175. Chronic Disease Prevention Unit, National Institute for Health and Welfare, Helsinki, Finland.
176. Department of Public Health, University of Helsinki, Helsinki, Finland.
177. Saudi Diabetes Research Group, King Abdulaziz University, Jeddah, Saudi Arabia.
178. Department of Internal Medicine, Erasmus MC, Rotterdam, the Netherlands.
179. Research Institute for Primordial Prevention of Non-communicable Disease, Isfahan University of Medical Sciences, Isfahan, Iran.

180. Interfaculty Institute for Genetics and Functional Genomics, University Medicine Greifswald, Greifswald, Germany.
181. Department of Internal Medicine, University Hospital, CHUV, Lausanne, Switzerland.
182. Experimental Genetics Division, Sidra Medical and Research Center, Doha, Qatar.
183. Centre for Population Health Sciences, Usher Institute of Population Health Sciences and Informatics, University of Edinburgh, Scotland, UK
184. Department of Biology, Faculty of Medicine, University of Split, Croatia.
185. The National Institute for Health Research Blood and Transplant Research Unit in Donor Health and Genomics, University of Cambridge, UK.
186. Division of Cardiology, University Hospital, Basel, Switzerland.
187. Division of Cardiology, Department of Medicine, McMaster University, Hamilton, Canada.
188. Institute of Genetic and Biomedical Research, National Research Council (CNR), Monserrato, Cagliari, Italy.
189. Department of Biomedical Sciences, University of Sassari, Sassari, Italy.
190. Institute of Clinical Medicine, Internal Medicine, University of Eastern Finland and Kuopio University Hospital, Kuopio, Finland.
191. Laboratory of Cardiovascular Science, NIA/NIH , Baltimore, MD, USA.
192. Department of Public Health and Primary Care, Leiden University Medical Center, Leiden, the Netherlands.
193. Labormedizinisches Zentrum Dr. Risch, Schaan, Liechtenstein.
194. Private University of the Principality of Liechtenstin, Triesen, Liechtenstein.
195. University Insitute of Clinical Chemistry, Inselspital, Bern University Hospital, University of Bern, Bern, Switzerland.
196. Department of Cardiology, University of Groningen, University Medical Center Groningen, Groningen, The Netherlands.
197. Center for Genomic Medicine, Massachusetts General Hospital, Boston, MA, USA.
198. Cardiovascular Research Center, Massachusetts General Hospital, Boston, MA, USA.

201. Cardiovascular Health Research Unit, Departments of Medicine, Epidemiology and Health Services, University of Washington, Seattle, WA, USA.
202. Kaiser Permanente Washington Health Research Institute, Seattle, WA, USA.
203. National Institute for Health Research Imperial Biomedical Research Centre, Imperial College Healthcare NHS Trust and Imperial College London, London, UK.
204. UK Dementia Research Institute (UK DRI) at Imperial College London, London, UK
205. Health Data Research-UK London substantive site, London, U.K
